# Web Application for Quantification of Traumatic Brain Injury-Induced Cortical Lesions in Adult Mice

**DOI:** 10.1007/s12021-019-09444-9

**Published:** 2019-12-04

**Authors:** Robert Ciszek, Pedro Andrade, Jesse Tapiala, Asla Pitkänen, Xavier Ekolle Ndode-Ekane

**Affiliations:** grid.9668.1 0000 0001 0726 2490 A. I. Virtanen Institute for Molecular Sciences, University of Eastern Finland, PO Box 1627, FI-70211 Kuopio, Finland

**Keywords:** Web applications, Visualization, Traumatic brain injury, Mouse brain

## Abstract

Disabilities resulting from traumatic brain injury (TBI) strongly correlate with the cytoarchitectonic part of the brain damaged, lesion area, and type of lesion. We developed a Web application to estimate the location of the lesion on mouse cerebral cortex caused by TBI induced by lateral fluid-percussion injury. The application unfolds user-determined TBI lesion measurements, e.g., from histologic sections to a reference template, and estimates the total lesion area, including the percentage of cortex damaged in different cytoarchitectural cortical regions. The resulting lesion can be visualized on a two-dimensional map of mouse cerebral cortex. The application also visualizes the development of the lesion over time when measurements from multiple time points are available. The web application was validated by comparing its performance to the manual method. The total area of the cortical lesion was similar between the manual (9.19 ± 0.66 mm^2^, range 4.25–14.93 mm^2^) and the automated analysis (9.27 ± 0.66 mm^2^, range 4.50–15.10 mm^2^) (*p* = 0.938). The results of the manual and automated analyses were strongly correlated (r = 0.999, *p* < 0.0001, Pearson correlation). The lesion localized in the same cytoarchitectonic regions when the unfolded map from the automated method was superimposed onto the map obtained using the manual method. The Web application-automated method is faster than the manual method in generating unfolded cortical lesion maps. The accuracy of the presented automated method in determining the anteroposterior level and outlining the lesion is equal to or greater than that of the manual method. Our application provides a novel tool for accurately quantifying and visualizing TBI lesions on mouse cerebral cortex.

**Electronic supplementary material:**

The online version of this article (10.1007/s12021-019-09444-9) contains supplementary material, which is available to authorized users.

## Background

The localization of traumatic brain injury (TBI) lesions directly correlates with motor disabilities and cognitive impairment (Chen et al. 2000). Ndode-Ekane et al. (Ekolle Ndode-Ekane et al. 2017) demonstrated that by unfolding magnetic resonance (MR) images of a lesioned rat cortex on a two-dimensional (2D) cytoarchitecture map of the cortex, it is possible to predict the severity of the resulting cognitive and behavioral impairment. In the report by Ndode-Ekane and colleagues, translation of the cortical lesion measurements to an unfolded map of the cortex was performed semi-manually using a combination of digitized measurements and image processing software (Ekolle Ndode-Ekane et al. 2017). This process, however, including estimation of the area of the affected cytoarchitectonic regions, is time consuming. In addition, freehand measurements performed using typical image processing software result in inter-researcher variability. Such manual mapping procedure is computationally straightforward and can be automated.

Web applications are computer programs that run within a Web browser. Implemented essentially as dynamic Web pages, Web applications can be executed by simply accessing the Web address of the application. No software installation is required to run Web applications. Encapsulation of the executed code within the browser makes Web applications inherently multi-platform, enabling users to utilize the application on their operating system of choice (e.g., Microsoft Windows, iOS, or Linux).

In an earlier report, we demonstrated the use of a Web-application, unfolded map (http://www.unfoldedmap.org/), for the localization of cortical lesions in a rat model of TBI (Andrade et al. 2018). To facilitate research on TBI using mouse models, we have implemented a novel Web-application *CortexMap (**https://www.cortexmap.org**)* for localizing lesions in the mouse cerebral cortex. Using imported measurements from MR or histopathologic images, our tool automatically maps to a template of the unfolded mouse cortex and quantifies the lesion area as well as the percentage of the damaged area in the different cytoarchitectonic cortical regions. When multiple measurements of the same lesion from different time points are imported, CortexMap visualizes the evolution of the lesion as a downloadable video file. The tool was validated by comparing the output maps with those drawn manually. In addition to a Web-application, our tool is available as a command-line application for offline use. The command-line version has additional support for user-made templates and can be utilized as a part of, e.g., batch processing pipelines.

## Template of the Unfolded Cortex of the Mouse and Assessment of the Cortical Lesion

### Preparation of the Two-Dimensional (2D) Template of the Unfolded Mouse Cortex

**Fig. 1:**
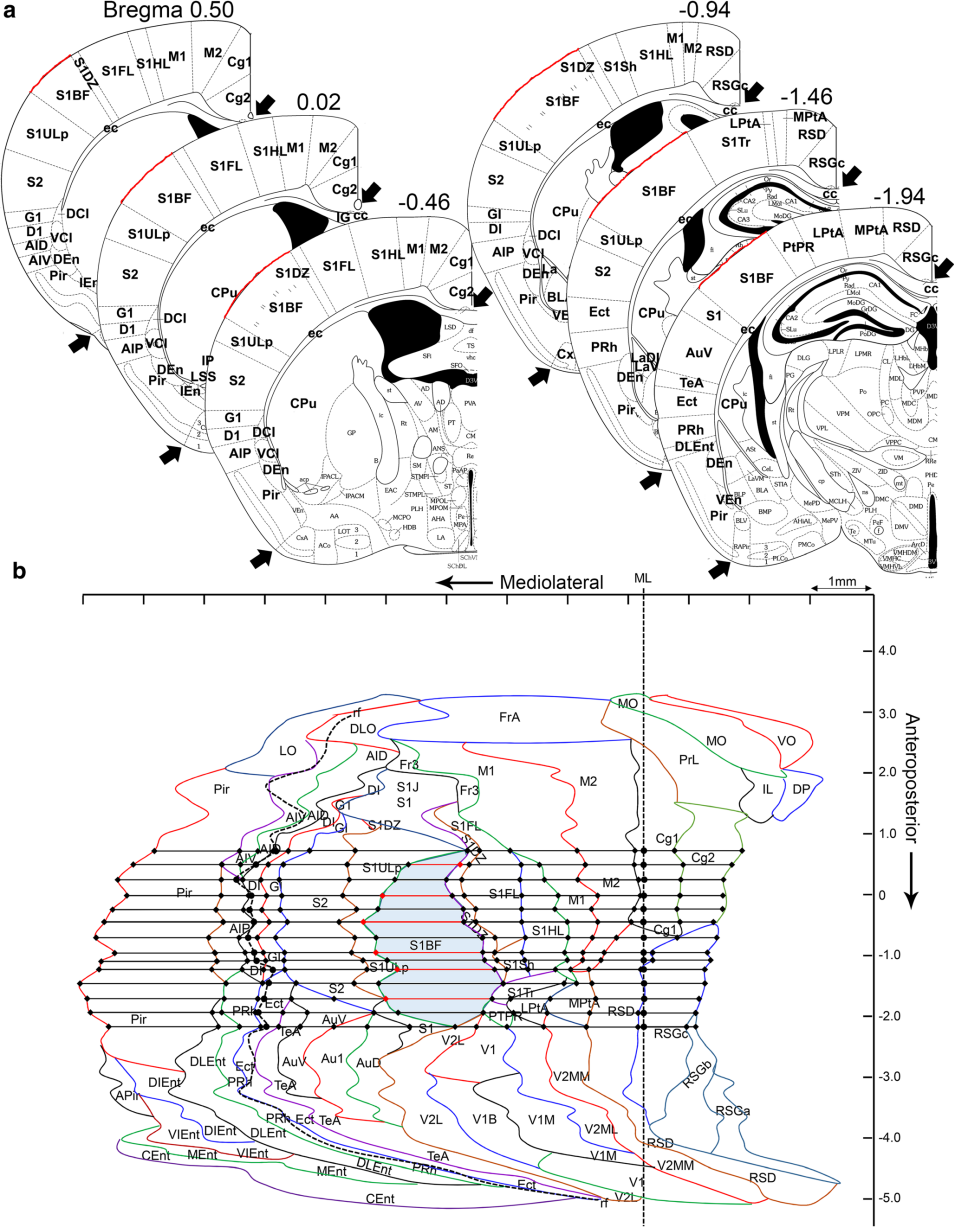
Template of the unfolded mouse cortex. Forty coronal plates between anteroposterior coordinates +3.20 to −4.96 with bregma as reference were used. On each atlas plate, the length of the outer boundary of each cortical region was measured along the brain surface, from the point of the midline where the cortex meets the corpus callosum (upper arrowhead) to the lateral edge of the piriform cortex (lower arrowhead). (**a**) Example of a measurement in the S1 cortex (red line) at different anteroposterior coordinates. (**b**) Measurement of the different cortical areas was transferred to a PowerPoint (see details in Methods). The points indicating the beginning and end of each cortical area on successive plates were connected to reveal the area boundaries as indicated here with the S1BF. The red line indicates measurements from the atlas plates in (A). Not that the rhinal fissure (rh) is depicted as dashed line and the Midline (ML)

The template of the unfolded map (UFM) of the mouse cerebral cortex used in the Web-application was created based on the technique previously described by (Van Essen and Maunsell 1980). The protocol for establishing the template was the same as that for rats previously described in Ekolle Ndode-Ekane et al. (2017) with minor modifications. The template was developed using the mouse brain atlas by Franklin (2007). In brief, 40 coronal plates (~200 μm between slices), between anteroposterior coordinates +3.20 to −4.96 with bregma as reference were used (see Fig. 1 for further details). The plates where converted to .tiff files and exported to ImageJ® software. Next, a lateral and a medial reference point was set along the pia surface in the different plates. In the anterior plates (bregma level + 3.20 to +2.10), the lateral reference point was the lateral edge of the *lateral orbital cortex*; in the plates between bregma level + 2.34 to −2.70, it was the lateral edge of the *piriform cortex*; in the plates between bregma −2.92 to −3.80, it was the lateral edge of the *amygdalopiriform transition area;* and in the most posterior plates (between bregma level − 4.04 to −4.96), it was the lateral edge of the *caudomedial entorhinal cortex*. The medial reference point was set at the intersection of the midline (ML) with the boundary between the cortex and the *corpus callosum*. In the anterior plates, where the *corpus callosum* is not present, however, we used either the medial edge of the *ventral orbital cortex* (+3.20 to +2.46) or the *dorsal peduncular cortex* (+2.10 to +1.34) and *cingulate cortex area 2* (+1.18 to +0.74). In the most posterior plates, the corpus callosum is also absent and thus we used the medial end of the cortex, which corresponds to the medial edge of the *rostrosplenial granular cortex* (−2.70 to −4.48) or the *retrosplenial dysgranular cortex* (−4.72 to −4.96). Following along the surface of the brain, we first measured the distance (in millimeters) between the medial and lateral reference points, and then the surface length of the different cortical regions in each coronal atlas plate. The measurements from the different cortical areas from each of the plates were transferred to a scaled PowerPoint slide using the line tool to draw straight lines (i.e., unfolded). Next, we connected the points representing the edges of the different cytoarchitectonic cortical areas to reveal the boundaries of these regions (for further detail, see) (Fig. 1). Furthermore, in every plate, we measured the distance between the medial reference point and the rhinal fissure. These values were later used to correct for tissue shrinkage during the manual or automated generation of the UFM (described below).

### Measuring the Cortical Lesion and Generating the Input Measurements

#### Generation of a Cortical Lesion Using the Lateral Fluid-Percussion Injury Model

The cortical injury was induced in adult male mice (C57BL/6JOlaHsd background) using the lateral fluid percussion injury model as previously described by Bolkvadze and Pitkänen (2012). In brief, a 3-mm diameter craniectomy was made in the skull of isoflurane-anesthetized mice between bregma and lambda on the left convexity, ensuring that the dura remained intact. The brain injury was induced by a transient fluid pulse impact (1.8 ± 0.1 atm, corresponding to severe injury) against the exposed dura using a fluid-percussion device (AmScien Instruments, Richmond, VA, USA). Following the impact, the dura was confirmed to be intact and the surgical site was sutured. The mice were administered buprenorphine (Temgesic® 0.05 mg/ml, s.c.) and saline (0.3 ml, s.c.), and allowed to recover in their home cage. At 30 d after the injury, the mice were sacrificed by transcardial perfusion with 4% paraformaldehyde. The brains were extracted and then processed for histology. The brains were cut into 25-μm thick sections in the coronal plane using a sliding microtome.

#### Generating Input Measurements for the UFM

Producing the UFM of the cortical lesion requires the location and extent of the lesion as input estimates. These estimates can be derived from histopathologic coronal brain sections or from coronal brain slices of MR imaging (MRI) volumes. For each histologic section or MRI slice, the medial reference point was identified as described above. In addition, the *rhinal fissure was* used as the lateral reference point. The distance from the medial reference point to medial edge of the lesion (**M1**), from the medial edge of the lesion to the lateral edge of the lesion (**M2**), and from the lateral edge of the lesion to the *rhinal fissure* (**M3**) was assessed (see Fig. 5 for details). Lesions in cortical layer V were used as a reference for defining the medial and lateral edges of the lesion. Additionally, the anteroposterior coordinate of each histologic section or MRI slice is noted (**MB**). If the lesion extends lateral to the rhinal fissure, *M*2 is measured from the medial edge of the lesion to the rhinal fissure, and *M*3 is measured from the rhinal fissure to the lateral edge of the lesion. For lesions extending lateral to the rhinal fissure, *M*3 is given a negative value. This approach allows for determining the mediolateral position of the lesion using the rhinal fissure as a lateral reference point. The measurements are stored in a comma-separated value (CSV) or Excel (.xls, .xlsx) file with four columns. The columns of the file present the measurements in the following order: *MB*, *M*1, *M*2 and *M*3. The UFM map automatically accounts for the voxel size of MR images by shifting the measurements along the anteroposterior axis by half of the voxel size on the corresponding axis.

## Software Description

### Calculating the Lesion Mapping

Lesion mapping is computed in four steps (**See supplementary Fig.** **2**): **1) normalization** of the estimate to account for differences in brain size between the atlas-based template and the experimental case, **2) translation** of the adjusted *M*3 and *M*2 distances to lesion coordinates on the template coordinate system, **3) interpolation** of the lesion contour, and **4) quantification** of lesion area and affected cytoarchitectonic cortical regions.

1. **Normalization** is performed separately for each slice. The inter-reference point distance (from medial reference point to the rhinal fissure) on the experimental case is calculated. If the lateral edge of the lesion is located on medial side of the rhinal fissure, the inter-reference point distance is the total sum of the *M*1, *M*2, and *M*3 distances. The inter-reference distance is divided by the cortical surface length in the atlas slice at the given anteroposterior level, which yields the ratio between the measured and atlas distances. The *M*2 and *M*3 distances are then multiplied by the ratio. *M*1 is omitted because the two first distances are sufficient for localization of the lesion. If the lesion extends to the lateral side of the rhinal fissure, the inter-reference point distance is the sum of *M*1 and *M*2. The ratio of this inter-reference point distance to the corresponding distance presented in the atlas is then calculated and used to adjust *M*3 and *M*2.
2. **Translation**. Distances *M*1, *M*2 and *MB* are converted into a lesion polygon in the template coordinate system using the template-specific units per millimeter ratio *r*. For each slice, lesion coordinates (r*M*1, *rMB*) and (*rM*2, *rMB*) are calculated by multiplying the lesion measurements by *r*. The lesion polygon is combined anticlockwise from all (*rM*1, *rMB*) pairs added in ascending order of *rMB*, followed by (*rM*2, *rMB*) pairs added in descending order of *rMB*.
3. **Interpolation** of the lesion contour is performed to estimate the mediolateral location of the lesion between the slices. For interpolation, either linear interpolation or Catmull-Rom spline interpolation (Catmull and Rom 1974) is used.
4. **Quantification**. The area of the intersection between each cytoarchitectonic region and the lesion contour is calculated. The region’s area of intersection is divided by the total area of the region to estimate the percentage of the region affected. The total area of cortex affected is the ratio of the sum of all affected areas to the total cortex area.

### Software Implementation

*CortexMap* is implemented using *Hypertext Markup Language revision 5* (*HTML5), Scalable Vector Graphics (SVG)*, *Canvas* application programming interface (API), *Web Workers*, *JavaScript* (ECMAScript 6), and *CSS3*. *Bootstrap* front-end framework and *jQuery* JavaScript library are utilized to construct the graphical user interface (GUI). The command-line interface (CLI) is executed using Node.js runtime within a headless Chromium instance. MediaStream Recording API is utilized to perform encoding of the lesion progression video within the browser.

**Fig. 2:**
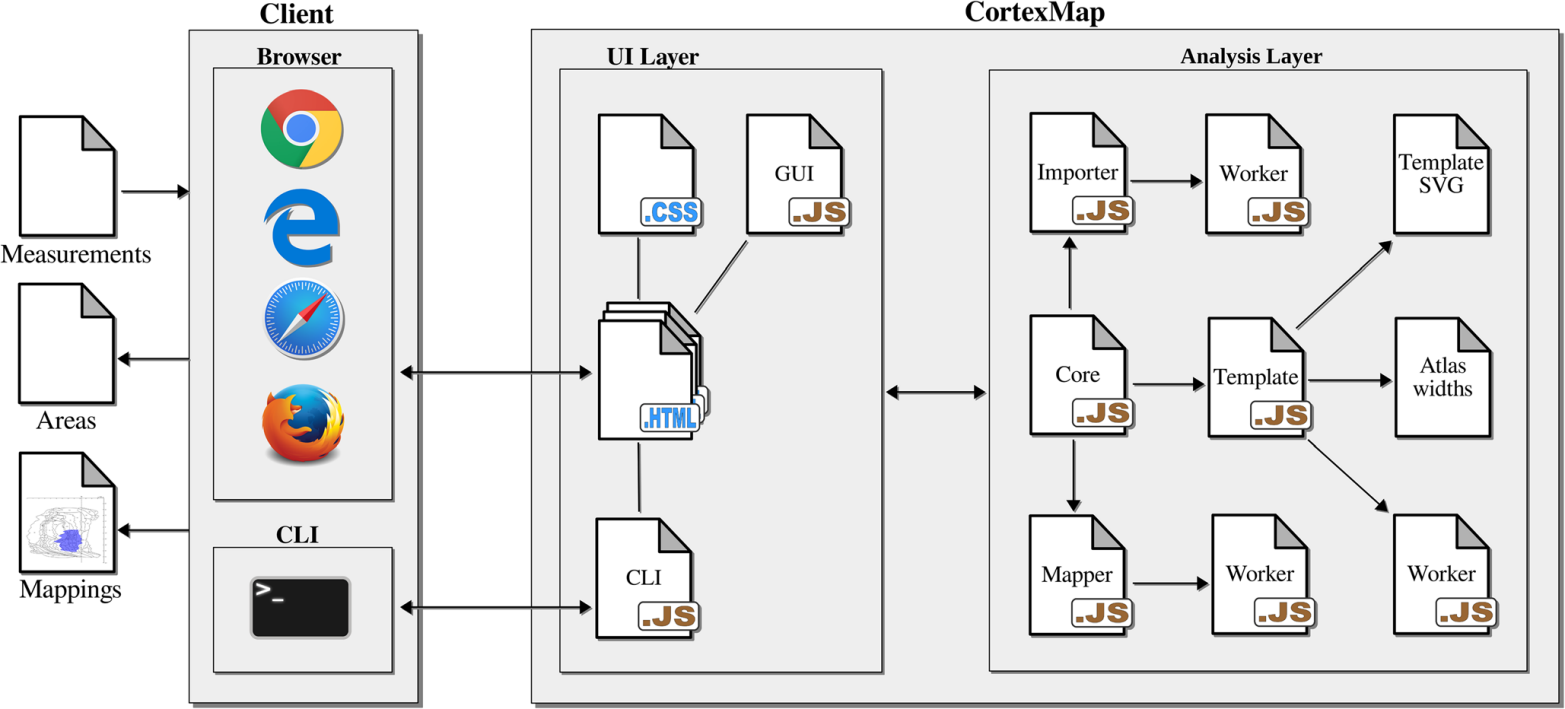
Software architectural division behind CortexMap. Components of the analysis layer are separated from the presentation logic contained within the UI layer. The core module functions as a façade to the functions of the analysis layer, forwarding method calls from the UI layer to the Importer and Mapper modules. The Importer, Mapper, and Template modules delegate time-consuming operations to WebWorkers using worker scripts

On a software architectural level (Fig. 2), the application is divided into a user interface (*UI) layer* and *analysis layer*. The *UI layer* is composed of HTML files, CSS file, the *GUI* JavaScript module, and CLI JavaScript file. The UI layer is responsible for presentation of the application and the handling of user input. The *GUI* module is decoupled from the rest of the system via a facade pattern implementing the Core module, which resides in the analysis layer. The Core module provides a simplified interface to the analysis procedure and reduces dependencies between the UI and the rest of the system.

The Core module communicates with the Importer, Mapper, and Template modules. Importer performs interpretation of coordinates presented in different file formats. The input files are read by executing a separate WebWorker thread. The Mapper module executes the mapping of the lesion using the Template module, which encapsulates the data derived from the template SVG file. Similarly, to the Importer module, the Mapper module performs the mapping of the given measurements in a dedicated WebWorker thread. In addition to functions for mapping the region of interest coordinates, the Mapper module also exposes functions for drawing the mapped region of interest on a given HTML5 Canvas for exporting the mappings as .tiff images.

The reference cortical surface lengths from the atlas are stored as a JSON file. A plain SVG file is used to store the template data. SVG images are defined as XML text files, enabling queries on the contents of the images, e.g., to enumerate the different objects presented in the image. Each cytoarchitectonic region of the template is presented in the SVG image using a unique closed path, labeled with prefix “A_”. The zero point of the anteroposterior axis is presented by a rectangle labeled “ap_zero”, in which the y position denotes the zero point. Alternatively, the zero point can be denoted by a similarly named attribute of the image’s svg element. The template specific units per millimeter constant can likewise be presented either by a rectangle labeled “units_per_mm”, with width equal to the units per millimeter in the template space, or by an attribute of the svg element with the same name. When both attributes and svg rectangles are included in the same file, precedence is given to attributes over rectangles. Location of the rhinal fissure on the template is denoted by a path labeled “rhinal_fissure”. The SVG file can contain additional SVG objects for visualization purposes, e.g., area labels or axis lines. The additional objects will be included in the visualization of the map, but are omitted during calculations.

During application initialization, the SVG paths-presenting region areas in the image are transformed to polygons by approximating the paths’ cubic Bezier curves using Casteljau’s subdivision method (de Casteljau 1959). Using the polygon of the interpolated lesion contour and the polygons denoting cortical regions, the area calculations are implemented as Boolean operations on polygons.

The transition between two lesions in a time series data set is visualized using linear interpolation. When two subsequent measurement differ in the number of slices, dummy points are added on the contour of the lesion with less slices. The points are removed after the transition visualization is completed.

### User Interface and Workflow

**Fig. 3:**
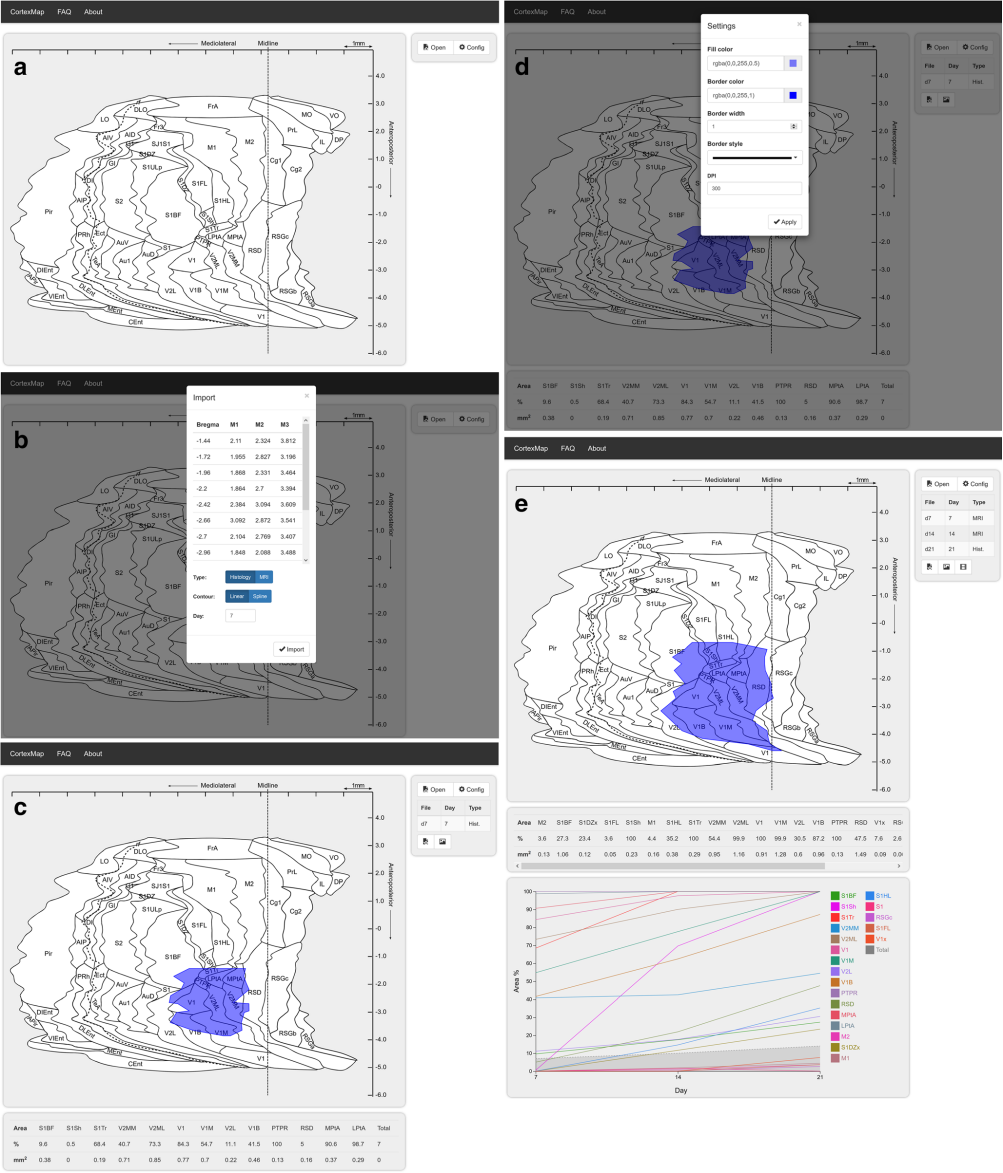
User interface. **(a)** Initially a plain map with buttons for opening a measurement file and changing the image configuration are displayed. **(b)** Measurements from an opened file and mapping parameters are displayed in a separate modal window. **(c)** The mapped lesion is displayed over the unfolded cortex. **(d)** The estimated affected areas are displayed in the table below the map. Visualization settings can be adjusted from the settings modal window. **(e)** When time series of measurements is imported, the percentage of areas affected at different time points is visualized as a chart below the ratio table

The import button in the upper right UI column (Fig. 3a) opens a file selection dialog. The contents of the selected coordinate file and available mapping settings are displayed in an import modal window (Fig. 3b). From the mapping settings, the user can select between mapping o*f histopathologic images* and *MR images*. For MR images, *slice depth* must be specified. For interpolation, either *linear* or *spline* interpolation can be used. The spline interpolation requires the user to define the number of points inserted between the existing measurements to create the spline. A large number of points results in a smooth interpolation, whereas a small number of points results in a jagged contour. The measurement day (day one by default) is defined in the bottom of the import modal. The measurement day defines the time point of measured lesion in a time series of measurements from different days.

Mapping is performed by pressing the “Import” button, visible on the lower part of the import modal window (Fig. 3b). The estimated contour of the lesion is visualized on the map image displayed on the interface (Fig. 3c). The area quantification results are displayed in a table below the map image. (Fig. 3c). For each affected cytoarchitectonic cortical region, the percentage and area (mm^2^) of the region affected by the lesion are presented. The total percentage and area (mm^2^) of cerebral cortex affected are displayed at a separate column in the rightmost end of the table.

After first measurement file has been imported, a table presenting the imported measurement file will appear. Below the table, buttons with file and image icons are displayed. The former allows the user to export the quantification results in a CSV format. The latter enables the displayed lesion mapping to be exported in tagged image file(TIFF) and in portable document file (PDF) formats. The mapping is rendered in both formats, and the resulting files are compressed into a ZIP-file for download.

When measurements from two or more days are imported, an interactive chart presenting the percentage of areas affected at different time points is presented below the ratio table (Fig. 3e**, See supplementary materials for details**). The chart is included in TIFF and PDF formats in the downloadable ZIP-file - along with the area mappings, when the image export button is clicked. A visualization of the evolution of the lesion over the time points can be downloaded in WebM format by clicking the button with video icon, which is visible when measurements from two or more days are mapped. Imported measurements can be deleted by selecting a measurement from the measurement table, and clicking a button with trashcan icon which appears next to the video download button when a measurement is selected.

**Fig. 4:**
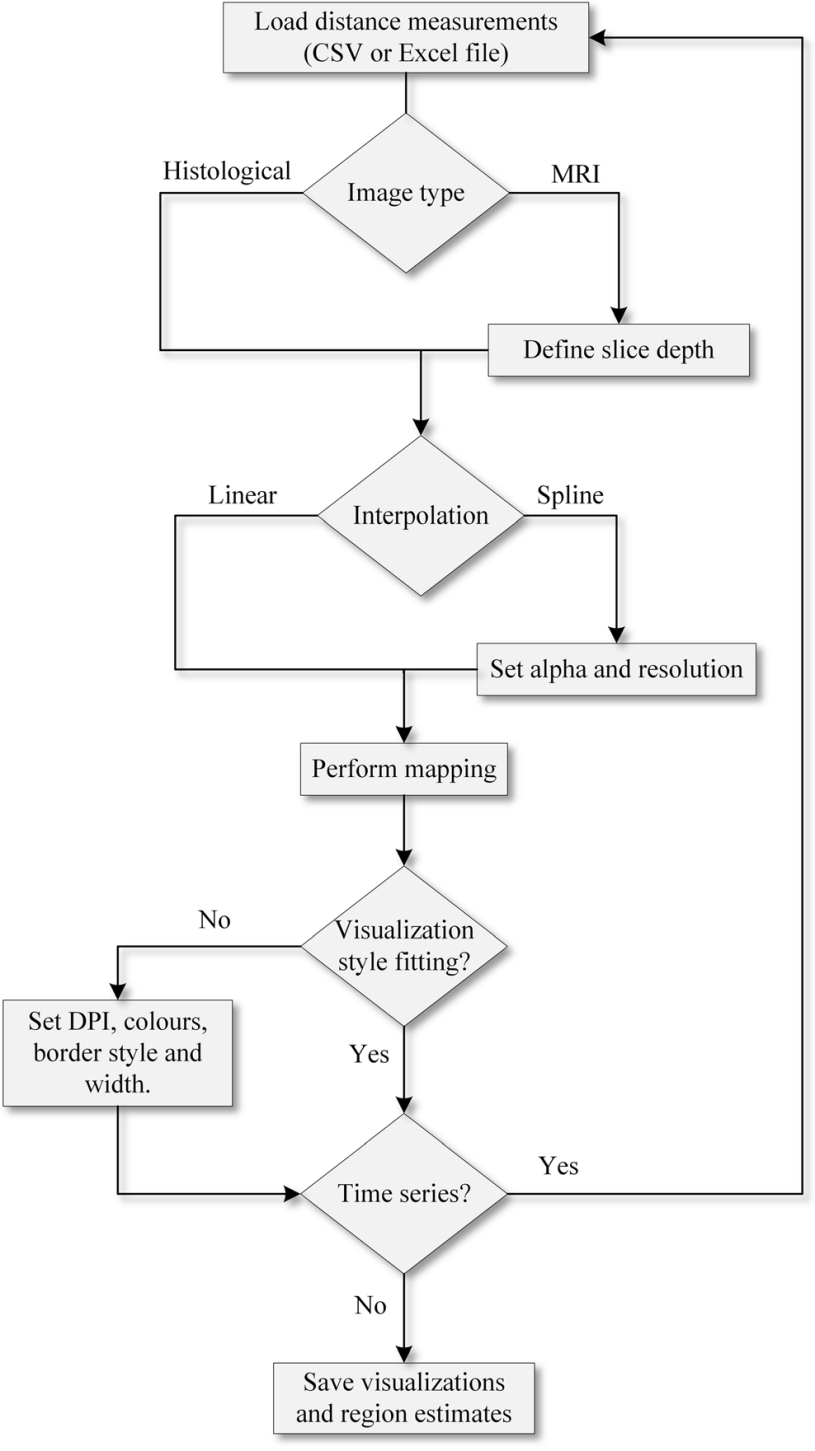
Lesion mapping workflow using the CortexMap Web-application. The user imports the lesion measurements by loading, e.g., a CSV file. For magnetic resonance images, the voxel size on the anteroposterior axis must be specified. The contour of the mapped lesion can be interpolated either linearly or by using Catmull-Rom spline. For spline interpolation, alpha and spline resolution must be specified. The resulting map can be saved as TIFF image and the region estimates can be saved as a CSV file

From the settings menu opened by the button at the upper right corner of the interface, the user can set the color and transparency of the mapped lesion (Fig. 3d). The lesion border color and the lesion area fill color can be defined separately. Either a solid, dashed, or dotted line style can be selected for the lesion border. From the configuration modal window, dots per inch of the downloadable result image can also be adjusted. The overall mapping workflow using the CortexMap Web-application is presented in Fig. 4.

The CLI provides all functionalities present in the Web-application, but with added configurability. Through the CLI, the user can either process a single measurement file or all measurement files contained within a specific folder. The user can additionally specify a custom cortex template to be used. The command-line parameters for the CLI version of CortexMap are presented in **supplementary Table 3.**

### Accessing the Software

CortexMap is located at URL *www.cortexmap.org* and is executed within the browser on the user’s workstation. During execution, both the measurements and the resulting maps are retained within the user browser. No communication occurs between the browser and the remote server after the application has been loaded. No browser plugins (e.g., *Adobe Flash Player* or *Java)* are required to execute the application. After page refresh or browser shutdown, mapping results are lost unless specifically exported from the application by the user.

The CLI version of the application can be installed through Node.js package manager, by typing command *npm install cortexmap-cli* in a folder selected by the user. After installation, the CLI application can be executed with command *cortexmap-cli*.

## Software Validation

### Materials & Method

#### Manual Unfolding of the Cortical Lesion from Histologic Sections

**Fig. 5:**
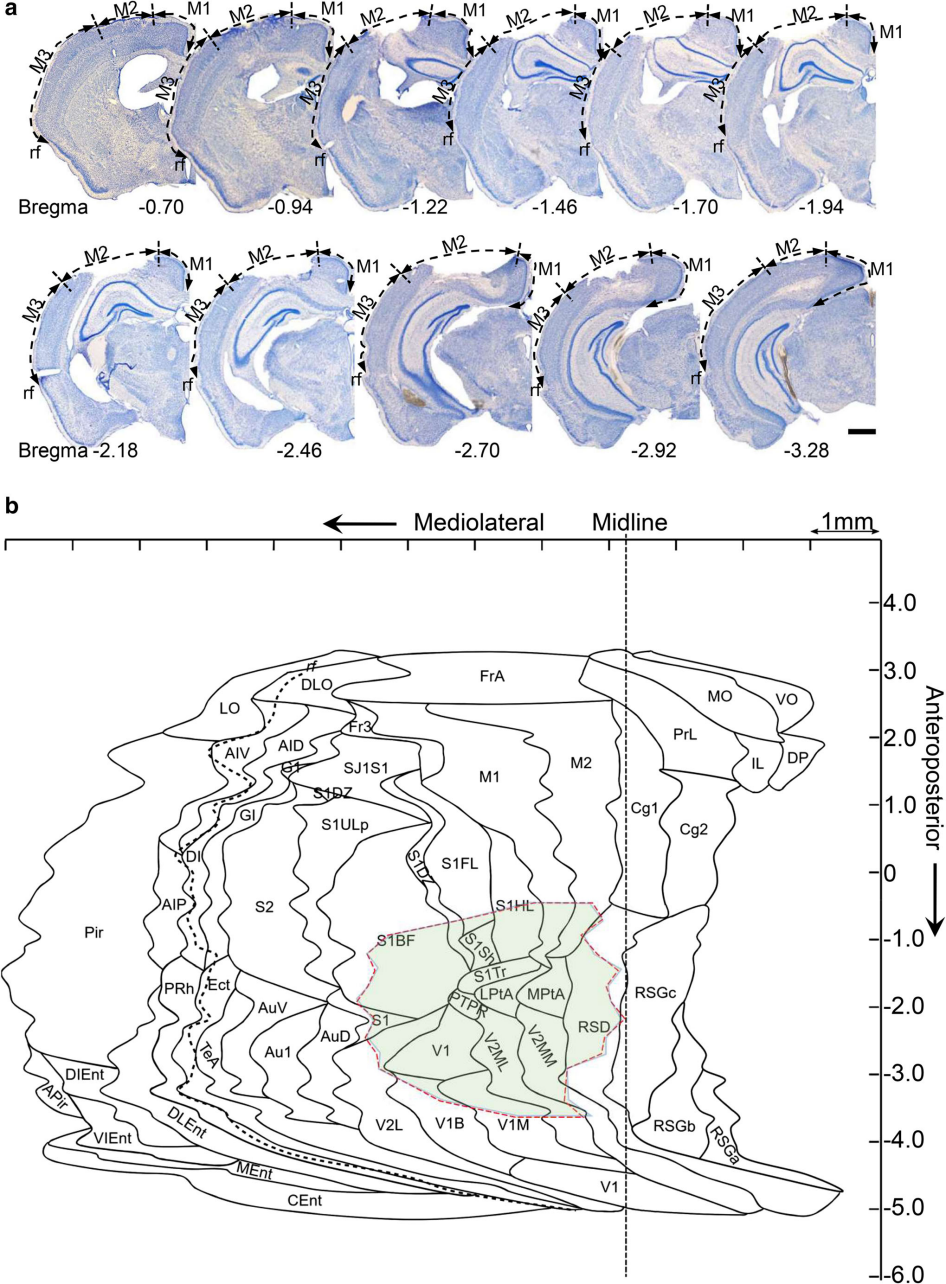
Parameter measurements and representative unfolded map (UFM) of a mouse cortext. (A) Demonstration of M1, M2, and M3 on a thionin-stained histologic section from a mouse (case #329) with lateral fluid percussion brain injury. (B) Representative UFM generated from case #329 using the automated Web application (red dashed line) superimposed on the manually generated map (light green shading). Measurements for M1, M2, and M3 are provided in supplementary Table 1. Horizontal line (medio-lateral) tick marks equal 1 mm distance. Vertical line (rostro-caudal) is shown with 0.1 mm accuracy (bregma equals 0). Scale bar in A = 1 mm

The cortical lesion following lateral fluid percussion injury was evaluated using the unfolded map technique previously described by (Ekolle Ndode-Ekane et al. 2017). The cortical lesion was evaluated from thionin-stained 25-μm thick coronal brain sections (300 μm apart from each other) in mice killed at 30 d following TBI. First, panoramic images of each coronal section were captured using the Zeiss Imager M2-microscope (Carl Zeiss AG, Germany). The images were exported as .tiff files into ImageJ software. The medial reference points were identified as described above. For the lateral reference point, we use the rhinal fissure. Next, along the surface of the brain, we measured the distance from the medial reference point to medial edge of the lesion (M1), then from the medial edges of the lesion to the lateral edge of the lesion (M2) and from the lateral edge of the lesion to the rhinal fissure (M3) (Fig. 5a). The lesion in cortical layer 5 was used as the reference for defining the medial and lateral edges of the lesion. Tissue shrinkage was estimated by calculating the ratio of the distance between the medial and lateral reference points in the section to that of the atlas in same anteroposterior coordinate. The measurements from each section (M1, M2 and M3) were then transferred to the template using the PowerPoint line tool at the exact anteroposterior coordinate (MB). The lesion was then revealed by connecting the edges of the lesion (L2) in each section (Fig. 5b) (for details, see (Ekolle Ndode-Ekane et al. 2017)).

#### Quantification of the Lesion Area from the Manually Generated Unfolded Map

The unfolded cortical lesion maps (.tiff files) were opened in ImageJ. The spatial scale was then set in ImageJ to match the scale of the unfolded map (Analyze / Set scale). The lesion was selected using the polygon selection tool and the area was calculated automatically (Analyze / Measure). The area of the lesion falling within each of the different cytoarchitectonic cortical areas was calculated in a similar fashion.

#### Procedure for Automatic Unfolding of the Cortical Lesion Map Using the UFM Web-App

1. Obtain images of the brain from histologic sections or MRI slices and assess the lengths of M1, M2, and M3 as described above. For details on how to obtain the measurement in MR images see Andrade et al. (2018) and Ekolle Ndode-Ekane et al. (2017).
2. Enter the measured values in Excel columns in the following order: MB (anteroposterior atlas coordinate), M1, M2, and M3, and save the files. Where the lesion extends lateral to the rhinal fissure, enter M3 as a negative value (see above).
3. Using the Web browser, open the URL www.cortexmap.org
4. Open a file selection dialog by pressing the “Open” button.
5. The contents of the selected coordinate file and available mapping settings are displayed in the import modal window.
6. Select the type of preparation used: “Histology” for histologic sections and “MRI” for magnetic resonance images. For MR images, *slice depth* must be specified.
7. Choose between *linear* interpolation or *spline* interpolation. For spline interpolation, set the parameter alpha and the number of points used for smoothing by the spline.
8. If the measurements are part of a time series, define the measurements day in the “Day” box at the bottom of the modal. If not, the default value of day 1 can be used.
9. Press the “Map” button to perform the mapping.
10. The lesion is visualized on the map displayed on the interface. The table of affected regions is presented as a table below the cortex map.
11. If measurements from more than one day are to be mapped, return to step 4 and import the remaining measurements.
12. To alter the visualized lesion, press the “Settings” button. From the opened settings modal window, set the lesion border color and the lesion fill color. Choose between solid, dashed, or dotted border styles. Increase or decrease the lesion border width to achieve the desired width. Set the dots per inch of the resulting image.
13. Press button with file icon to export the ratios of the affected regions of the cortex in a CSV file. Press the button with image icon to export the mapped lesions TIFF and PDF. If measurements from more than one day are imported, click the button with video icon to export a video of the lesion development.

### Results and Validation

#### Cytoarchitectonic Location of the Lesion on the Cortical Mantle: Manual Method Vs. Web Application

A total of 18 cases were used to validate the Web application. In all cases, when the 2D UFM generated with the manual method was superimposed onto that generated by the automated method (Web application), the lesion was observed to colocalize on the same cytoarchitectonic areas (Fig. 5b, **see also supplementary Fig. 2 and Table 1**). There were minimal deviations, however, which were most likely due to human errors resulting from imprecise plotting of the anteroposterior coordinate. The automated method can precisely plot the manually determined anteroposterior coordinate on the template with 0.01 mm precision as compared to the manual method.

#### Area of the Cortical Lesion: Manual Method Vs. Web Application

**Fig. 6:**
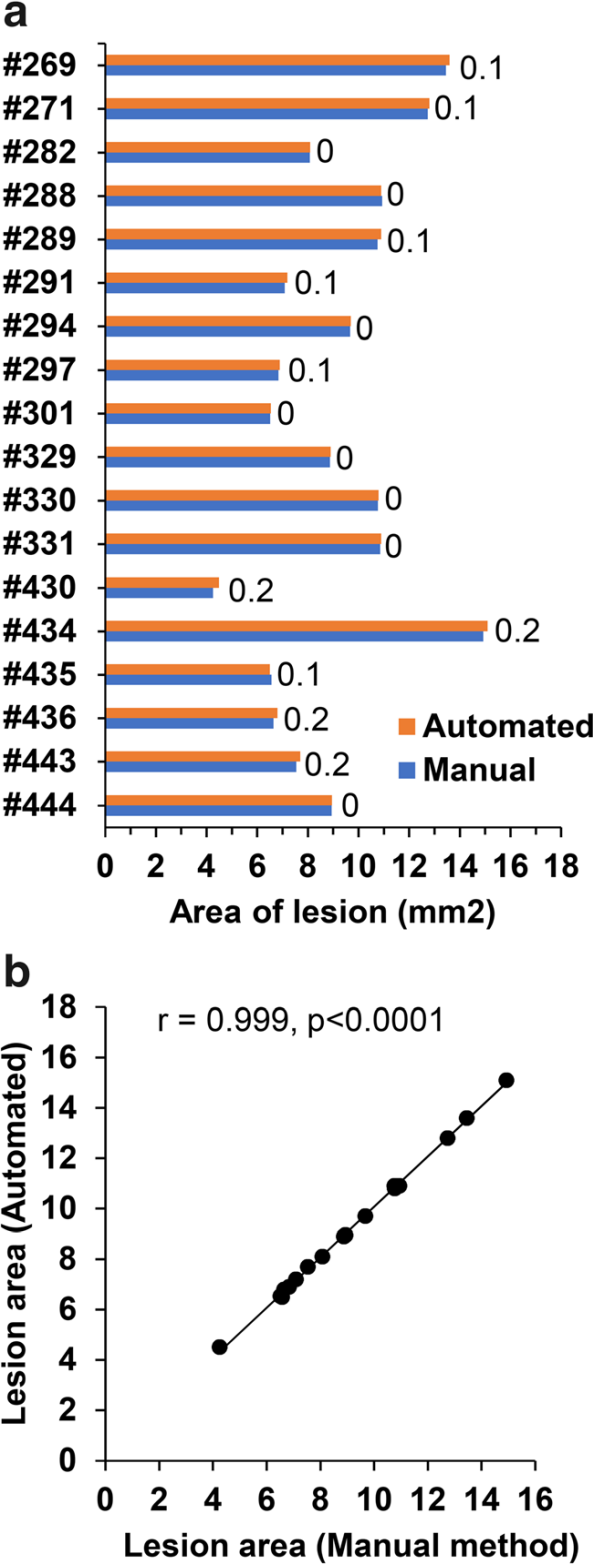
Bar graph and scatter plot of the lesion area generated manually and by Web application (automated method). **(a)** Bar graph showing the area of the cortical lesion estimated from the unfolded map generated by the manual method and the Web application method (automated). Numbers in the graph indicate the difference in the lesion area between methods. Note the minimal difference between the two methods **(b)** Scatter plot showing the correlation between the manual and automated methods (Pearson correlation)

The total area of the lesion did not differ between methods: 9.19 ± 0.66 mm^2^ (range 4.25–14.93 mm^2^) in the manual method and 9.27 ± 0.66 mm^2^ (4.50–15.10 mm^2^) in the automated method (*p* = 0.938, Mann Whitney *U* test, mean ± SEM) (Fig. 6a). In the individual cases, the difference in the total area between the manual and the automated method ranged from −0.2 mm^2^ to 0.1 mm^2^ (**see also** Fig. 6a). Additionally, the total area of the different cytoarchitectonic areas was compared between maps generated using both the manual method and the automated method (see **supplementary Table 2**). Correlation analysis revealed a strong correlation between the manual and automated methods (r = 0.999, *p* < 0.0001, Pearson correlation test) (Fig. 6b). The difference between methods may be due to human error in plotting the measurement at the exact anteroposterior coordinate.

## Discussion

We developed a user-friendly application for automated 2D mapping of cortical lesions measured from histopathologic or MR images to a 2D template of unfolded mouse cortex. The application enables accurate and fast quantification of the lesion area and the affected cytoarchitecture regions. The main advantages of *CortexMap* are:

1. **Repeatability**: The same input measurements produce the exact same map for each researcher, removing inter-researcher variance.
2. **Accuracy**: Compared with the manual method, the Web application has equal or greater accuracy.
3. **Speed**: Automated generation of unfolded cortical maps of lesioned cortical areas is approximately 60 times faster than manual mapping. That is, it takes approximately 30 min to measure M1, M2, and M3 from digital images of histologic sections (11–13 sections) or MRI and enter the data into Excel. It takes approximately 1 h to manually map or plot the measurements onto PowerPoint slide with the template to produce the lesion. On the other hand, it takes less than a minute to map or plot the lesion using the automated Web application.
4. **Accessibility**: The Web application can be accessed everywhere without restrictions or need for prior installation. No installation of third-party browser plugins is necessary to execute the application. For offline use, the application can be installed through Node.js package manager (npm).

We have previously reported a similar Web app for the identification of the cortical lesion in the rat brain following TBI (Andrade et al. 2018). The mouse version presented in this study is operated the same as the rat version. However, the mapping back-end and the user experience with the GUI have received significant novel additions including the possibility to generate and visualize lesion progression from same animal using time series data acquired for example with MRI. This will significantly enhance follow-up of lesion progression. Other new features such as batch analysis using CLI will enable user to perform more maps in a shorter time.

In the current study the application was used for mapping the cortical lesion in mouse with severe TBI. The application can be extended to mild and moderate TBI mouse models and can also support other animal models. The limiting factor in the mapping is how the user defines the lesion boundaries when using histological or MR images. In future work, we intend to provide templates for utilization of the presented method in experiments performed, e.g., on monkeys. We will also add functionality for the visualization of lesion penumbra as a heatmap.

## Limitations

The web application accurately maps the manually determined measurements on to the template. Therefore, incorrect identification and measurement of the lesion boundaries on histological or MR images will result in a wrong cytoarchitectonic map of the lesion. It is therefore important that the user is competent in identifying the lesion in histological sections or MR images. In this study we use the cortical layer V to map the lesion boundaries. Due to our long-standing experience with this TBI model, we realized that Layer V is usually the most damaged layer. However, this is not always the case in some animals, thus, using layer V in this instance can lead to either an over estimation or underestimation of the lesion size. Another limitation is the difficulty in identifying layer V in MR images. Despite these limitations, we have previously shown that it is possible to identify the cortical lesion in MR images to the same cytoarchitectonic areas as in histologic sections using the unfolded map technique (Ekolle Ndode-Ekane et al. 2017). Other aspects that may compromised the mapping of the lesion with the web application including brain size, tissue shrinkage and accurate mapping of the anteroposterior coordinate have been previously published. For detail see Ekolle Ndode-Ekane et al. (2017).

## Information Sharing Statement

The source code of CortexMap is available at https://github.com/UEFepilepsyAIVI/CortexMap. The CLI version of the application is available through npm with the name cortexmap-cli.

## Electronic supplementary material

ESM 1 (DOCX 89 kb)

ESM 2 (PPTX 11813 kb)

ESM 3 (PDF 9600 kb)

ESM 4 (WEBM 1866 kb)
