# Supplementary material for: Web Application for Quantification of Traumatic Brain Injury-Induced Cortical Lesions in Adult Mice
Source: Neuroinformatics. 2019 Dec 4;18(2):307–17. doi: 10.1007/s12021-019-09444-9 (PMC7083813; doi:10.1007/s12021-019-09444-9)
Supplement: Supplementary file 1 — (DOCX 89 kb) [file 12021_2019_9444_MOESM1_ESM.docx]

| **Supplementary Table 1A.** M1, M2 and M3 measurement from 6 mice. The unfolded maps generated from the measurements are shown in Supplementary **Figure 1**. Bregma levels (MB) are defined according to the mouse brain atlas by Watson and Paxinos (2007). | | | | | | | | | | | | | | | | | | | | | | | |
| --- | --- | --- | --- | --- | --- | --- | --- | --- | --- | --- | --- | --- | --- | --- | --- | --- | --- | --- | --- | --- | --- | --- | --- |
| **#269** | | | | **#271** | | | | **#282** | | | | **#288** | | | | **#289** | | | | **#291** | | | |
| **MB** | **M1** | **M2** | **M3** | **MB** | **M1** | **M2** | **M3** | **MB** | **M1** | **M2** | **M3** | **MB** | **M1** | **M2** | **M3** | **MB** | **M1** | **M2** | **M3** | **MB** | **M1** | **M2** | **M3** |
| 0.74 | 3.081 | 1.182 | 3.716 | 1.18 | 2.728 | 1.185 | 3.603 | 0.26 | 3.043 | 1.105 | 2.136 | 0.74 | 1.89 | 2.124 | 3.532 | 0.86 | 2.651 | 0.851 | 4.555 | -0.7 | 3.142 | 1.183 | 2.807 |
| 0.5 | 2.192 | 2.886 | 2.768 | 1.1 | 1.798 | 1.833 | 3.716 | 0.02 | 3.179 | 0.955 | 2.999 | 0.5 | 2.409 | 2.13 | 2.767 | 0.5 | 2.307 | 2.226 | 3.621 | -0.94 | 1.505 | 3.235 | 2.952 |
| 0.26 | 1.877 | 2.012 | 4.188 | 0.98 | 2.428 | 2.247 | 3.473 | -0.34 | 2.11 | 1.807 | 3.458 | -0.46 | 1.658 | 3.921 | 2.397 | 0.26 | 1.374 | 2.868 | 3.536 | -1.22 | 1.353 | 3.189 | 2.139 |
| 0.02 | 1.754 | 3.124 | 2.669 | 0.38 | 2.31 | 2.805 | 3.438 | -0.58 | 2.229 | 2.154 | 2.825 | -0.82 | 1.665 | 2.849 | 3.043 | -0.34 | 1.357 | 2.943 | 3.474 | -1.46 | 1.269 | 3.75 | 1.818 |
| -0.46 | 0.876 | 4.051 | 2.866 | 0.14 | 1.664 | 3.333 | 2.788 | -0.94 | 2.132 | 2.163 | 3.089 | -1.06 | 1.47 | 3.22 | 2.357 | -0.82 | 1.221 | 3.048 | 3.03 | -1.58 | 1.107 | 4.095 | 1.666 |
| -0.7 | 0.781 | 4.137 | 2.579 | -0.94 | 1.635 | 3.846 | 2.374 | -1.34 | 1.867 | 2.998 | 2.652 | -1.46 | 1.342 | 4.345 | 1.239 | -1.22 | 1.076 | 3.571 | 2.727 | -1.82 | 1.088 | 3.953 | 1.866 |
| -1.06 | 0.632 | 4.177 | 2.286 | -1.22 | 1.434 | 3.987 | 2.575 | -1.58 | 1.738 | 2.85 | 2.727 | -1.7 | 1.259 | 4.177 | 1.608 | -1.46 | 1.031 | 3.742 | 2.484 | -2.06 | 1.261 | 3.878 | 2.048 |
| -1.34 | 0.546 | 5.303 | 1.689 | -1.46 | 1.481 | 4.141 | 1.608 | -1.7 | 2.062 | 2.679 | 2.724 | -1.94 | 1.418 | 4.102 | 1.406 | -1.7 | 1.022 | 4.235 | 1.94 | -2.18 | 1.657 | 3.849 | 2.142 |
| -1.7 | 0.274 | 4.821 | 2.137 | -1.7 | 1.542 | 3.893 | 1.869 | -1.94 | 1.992 | 2.801 | 2.038 | -2.06 | 1.413 | 3.876 | 1.535 | -1.94 | 0.849 | 3.814 | 2.475 | -2.3 | 2.028 | 3.348 | 2.705 |
| -1.94 | 0.287 | 4.734 | 2.905 | -1.94 | 1.371 | 4.484 | 1.766 | -2.18 | 2.269 | 3.057 | 2.407 | -2.3 | 1.463 | 3.765 | 1.607 | -2.06 | 0.993 | 4.018 | 2.317 | -2.46 | 2.076 | 3.559 | 2.733 |
| -2.18 | 0.232 | 4.817 | 2.78 | -2.18 | 1.606 | 4.184 | 2.065 | -2.46 | 2.38 | 2.97 | 2.442 | -2.46 | 2.518 | 3.074 | 2.446 | -2.3 | 1.372 | 4.077 | 1.827 | -2.54 | 2.004 | 2.675 | 3.218 |
| -2.46 | 0.574 | 5.058 | 2.425 | -2.46 | 2.243 | 4.639 | 1.827 | -2.7 | 3.156 | 2.433 | 2.686 | -2.54 | 2.69 | 2.343 | 3.01 | -2.46 | 1.383 | 3.478 | 2.881 | -2.7 | 2.113 | 2.255 | 3.334 |
| -2.54 | 0.915 | 4.46 | 3.028 | -2.7 | 2.717 | 2.154 | 3.634 | -2.92 | 2.662 | 2.295 | 3.095 |  |  |  |  | -2.54 | 1.459 | 2.928 | 3.158 | -2.92 | 2.09 | 1.472 | 3.786 |
| -2.7 | 2.082 | 3.123 | 3.407 | -2.8 | 2.563 | 0.898 | 4.426 | -3.16 | 3.005 | 2.072 | 2.934 |  |  |  |  | -2.7 | 2.318 | 2.033 | 4.092 | -3.16 | 2.101 | 1.272 | 3.815 |
| -2.8 | 2.456 | 1.916 | 3.986 |  |  |  |  | -3.4 | 4.161 | 0.894 | 2.62 |  |  |  |  |  |  |  |  |  |  |  |  |
| -3.08 | 3.835 | 0.815 | 3.455 |  |  |  |  | -3.52 | 4.524 | 0.776 | 2.493 |  |  |  |  |  |  |  |  |  |  |  |  |

| **Supplementary Table 1B.** M1, M2 and M3 measurement from 6 mice. Bregma levels (MB) are defined according to the mouse brain atlas by Watson and Paxinos (2007). | | | | | | | | | | | | | | | | | | | | | | | |
| --- | --- | --- | --- | --- | --- | --- | --- | --- | --- | --- | --- | --- | --- | --- | --- | --- | --- | --- | --- | --- | --- | --- | --- |
| **#294** | | | | **#297** | | | | **#301** | | | | **#329** | | | | **#330** | | | | **#331** | | | |
| **MB** | **M1** | **M2** | **M3** | **MB** | **M1** | **M2** | **M3** | **MB** | **M1** | **M2** | **M3** | **MB** | **M1** | **M2** | **M3** | **MB** | **M1** | **M2** | **M3** | **MB** | **M1** | **M2** | **M3** |
| -0.46 | 2.582 | 1.567 | 3.058 | -0.34 | 2.175 | 2.14 | 3.729 | -0.7 | 2.948 | 0.998 | 3.624 | -0.46 | 1.841 | 0.893 | 5.194 | -0.7 | 2.568 | 1.439 | 3.585 | -0.46 | 1.705 | 1.489 | 3.961 |
| -0.7 | 1.545 | 2.523 | 3.128 | -0.58 | 1.859 | 2.199 | 3.645 | -0.94 | 2.356 | 2.009 | 2.883 | -0.7 | 1.621 | 2.165 | 4.365 | -0.94 | 1.21 | 2.924 | 3.212 | -0.7 | 1.693 | 2.472 | 3.255 |
| -0.82 | 1.512 | 2.878 | 2.838 | -0.82 | 1.684 | 2.145 | 3.585 | -1.34 | 3.233 | 1.822 | 2.327 | -0.94 | 1.9 | 3.162 | 3.162 | -1.22 | 1.355 | 3.427 | 2.596 | -0.94 | 1.552 | 2.231 | 3.657 |
| -1.06 | 1.435 | 3.08 | 2.458 | -1.06 | 1.433 | 3.267 | 2.816 | -1.58 | 2.948 | 1.918 | 2.646 | -1.22 | 1.648 | 3.809 | 2.508 | -1.46 | 1.268 | 2.931 | 2.864 | -1.22 | 1.644 | 3.042 | 3.026 |
| -1.46 | 1.181 | 3.115 | 1.989 | -1.34 | 1.375 | 3.597 | 2.458 | -1.94 | 3.016 | 2.574 | 2.054 | -1.46 | 1.267 | 3.965 | 2.688 | -1.7 | 1.164 | 3.38 | 2.794 | -1.46 | 1.263 | 4.184 | 2.532 |
| -1.82 | 1.203 | 2.865 | 2.372 | -1.7 | 1.281 | 3.404 | 2.846 | -2.18 | 1.999 | 2 | 2.508 | -1.7 | 1.298 | 3.869 | 2.618 | -2.18 | 1.121 | 3.238 | 3.202 | -1.7 | 1.266 | 3.501 | 3.387 |
| -2.06 | 1.037 | 2.479 | 1.648 | -1.94 | 1.187 | 3.366 | 2.941 | -2.46 | 2.27 | 2.355 | 2.719 | -1.94 | 1.349 | 4.178 | 2.554 | -2.46 | 1.048 | 3.488 | 2.888 | -1.94 | 1.067 | 4.02 | 3.116 |
| -2.46 | 1.212 | 3.512 | 1.552 | -2.18 | 1.4 | 3.602 | 2.84 | -2.92 | 2.51 | 2.573 | 2.92 | -2.18 | 1.18 | 4.113 | 2.733 | -2.7 | 1.204 | 3.469 | 2.907 | -2.46 | 1.128 | 4.503 | 1.753 |
| -2.7 | 1.751 | 3.699 | 2.188 | -2.46 | 1.413 | 3.047 | 3.327 | -3.16 | 3.211 | 2.881 | 2.393 | -2.46 | 1.456 | 3.81 | 2.798 | -2.92 | 1.684 | 3.794 | 3.009 | -2.7 | 1.118 | 3.654 | 2.942 |
| -2.92 | 1.769 | 2.447 | 3.323 | -2.7 | 1.778 | 2.623 | 3.516 | -3.52 | 3.679 | 2.218 | 2.421 | -2.7 | 1.858 | 3.795 | 3.061 | -3.16 | 2.292 | 3.672 | 2.741 | -2.92 | 1.897 | 3.737 | 3.143 |
| -3.16 | 1.593 | 2.29 | 3.593 | -2.8 | 1.885 | 1.336 | 4.332 | -3.88 | 3.689 | 1.507 | 2.904 | -2.92 | 2.458 | 3.233 | 3.2 | -3.52 | 2.874 | 3.567 | 2.951 | -3.16 | 2.606 | 3.819 | 3.383 |
| -3.4 | 1.755 | 2.198 | 3.577 |  |  |  |  |  |  |  |  | -3.4 | 2.583 | 1.922 | 3.758 | -3.88 | 2.359 | 3.689 | 2.903 | -3.64 | 2.73 | 3.69 | 3.378 |
| -3.8 | 3.5 | 1.189 | 2.482 |  |  |  |  |  |  |  |  | -3.64 | 2.522 | 1.089 | 4.609 | -4.16 | 2.369 | 2.882 | 3.112 | -3.88 | 2.687 | 2.731 | 3.366 |
|  |  |  |  |  |  |  |  |  |  |  |  |  |  |  |  | -4.36 | 2.882 | 0.981 | 4.119 | -4.16 | 3.301 | 0.881 | 4.346 |
|  |  |  |  |  |  |  |  |  |  |  |  |  |  |  |  | -4.6 | 3.007 | 0.395 | 3.899 |  |  |  |  |

| **#430** | | | | **#434** | | | | **#435** | | | | **#436** | | | | **#443** | | | | **#444** | | | |
| --- | --- | --- | --- | --- | --- | --- | --- | --- | --- | --- | --- | --- | --- | --- | --- | --- | --- | --- | --- | --- | --- | --- | --- |
| **MB** | **M1** | **M2** | **M3** | **MB** | **M1** | **M2** | **M3** | **MB** | **M1** | **M2** | **M3** | **MB** | **M1** | **M2** | **M3** | **MB** | **M1** | **M2** | **M3** | **MB** | **M1** | **M2** | **M3** |
| -0.22 | 2.014 | 0.804 | 4.456 | 1.1 | 2.447 | 1.226 | 3.219 | 0.02 | 1.751 | 0.822 | 4.485 | -0.7 | 2.879 | 0.618 | 4.174 | -0.46 | 2.281 | 1.828 | 4.106 | 0.38 | 2.972 | 0.923 | 3.329 |
| -0.46 | 1.554 | 1.712 | 4.713 | 0.98 | 2.622 | 1.871 | 3.252 | -0.22 | 2.175 | 2.569 | 2.774 | -0.94 | 2.263 | 2.076 | 3.362 | -0.7 | 1.782 | 2.327 | 3.914 | -0.1 | 1.597 | 1.644 | 4.443 |
| -0.82 | 1.496 | 1.865 | 4.386 | 0.74 | 2.364 | 3.315 | 2.177 | -0.7 | 1.953 | 2.631 | 2.769 | -1.34 | 1.31 | 3.16 | 3.336 | -1.22 | 1.579 | 3.176 | 3.171 | -0.46 | 2.295 | 2.276 | 3.301 |
| -1.06 | 1.313 | 2.17 | 4.116 | 0.26 | 2.013 | 3.55 | 2.65 | -0.94 | 1.435 | 3.187 | 2.552 | -1.7 | 0.868 | 3.617 | 2.987 | -1.46 | 1.611 | 3.094 | 3.383 | -0.82 | 1.47 | 2.522 | 3.825 |
| -1.46 | 1.239 | 1.774 | 4.54 | -0.46 | 1.379 | 3.732 | 2.632 | -1.34 | 1.637 | 3.593 | 2.201 | -1.94 | 0.974 | 4.222 | 2.256 | -1.7 | 1.247 | 3.359 | 3.422 | -1.06 | 1.303 | 3.631 | 2.525 |
| -1.7 | 1.211 | 1.498 | 4.83 | -1.06 | 1.346 | 4.007 | 2.41 | -1.46 | 1.498 | 3.556 | 2.329 | -2.18 | 0.949 | 3.824 | 2.892 | -1.94 | 1.228 | 3.558 | 3.308 | -1.34 | 1.296 | 3.045 | 3.102 |
| -1.94 | 0.975 | 1.962 | 4.818 | -1.34 | 1.529 | 3.623 | 2.433 | -1.7 | 1.806 | 2.449 | 3.023 | -2.46 | 1.026 | 3.224 | 3.594 | -2.18 | 0.989 | 3.683 | 3.384 | -1.58 | 0.89 | 3.921 | 2.479 |
| -2.18 | 1.012 | 1.465 | 5.285 | -1.58 | 1.691 | 3.938 | 1.73 | -1.94 | 2.517 | 2.528 | 2.952 | -2.7 | 2.176 | 1.948 | 4.127 | -2.46 | 1.164 | 3.799 | 3.234 | -1.82 | 1.233 | 3.568 | 2.686 |
| -2.46 | 1.532 | 1.247 | 5.268 | -1.7 | 1.51 | 4.012 | 1.662 | -2.18 | 2.458 | 2.048 | 3.352 | -2.92 | 2.34 | 1.912 | 4.407 | -2.7 | 1.653 | 3.86 | 3.365 | -2.06 | 1.369 | 3.338 | 2.684 |
| -2.7 | 1.856 | 0.979 | 5.65 | -1.94 | 1.415 | 4.579 | 1.487 | -2.46 | 3.704 | 0.769 | 3.945 | -3.28 | 2.344 | 1.772 | 4.708 | -2.8 | 2.077 | 3.223 | 3.743 | -2.3 | 1.583 | 4.013 | 2.401 |
| -2.92 | 2.367 | 1.147 | 4.861 | -2.18 | 1.69 | 3.4 | 2.628 | -2.7 | 3.766 | 0.965 | 3.081 | -3.52 | 2.122 | 1.26 | 5.195 | -3.16 | 1.917 | 2.318 | 4.323 | -2.46 | 1.882 | 3.48 | 3.047 |
| -3.16 | 2.675 | 0.706 | 5.279 | -2.3 | 1.66 | 2.792 | 2.967 | -2.92 | 3.982 | 0.789 | 3.149 |  |  |  |  | -3.4 | 2.183 | 0.739 | 5.602 | -2.7 | 1.892 | 2.823 | 3.33 |
| -3.52 | 3.018 | 1.032 | 4.488 | -2.46 | 1.564 | 2.642 | 3.15 |  |  |  |  |  |  |  |  |  |  |  |  | -2.8 | 2.214 | 2.037 | 3.595 |
|  |  |  |  | -2.54 | 1.624 | 2.674 | 2.992 |  |  |  |  |  |  |  |  |  |  |  |  | -2.92 | 2.771 | 0.981 | 3.955 |
|  |  |  |  | -2.8 | 1.86 | 2.27 | 2.995 |  |  |  |  |  |  |  |  |  |  |  |  | -3.4 | 2.447 | 0.889 | 4.105 |
|  |  |  |  | -3.4 | 1.496 | 1.788 | 3.04 |  |  |  |  |  |  |  |  |  |  |  |  |  |  |  |  |
|  |  |  |  | -3.52 | 1.973 | 1.659 | 2.694 |  |  |  |  |  |  |  |  |  |  |  |  |  |  |  |  |
|  |  |  |  | -3.88 | 2.289 | 1.552 | 2.4 |  |  |  |  |  |  |  |  |  |  |  |  |  |  |  |  |

| **Supplementary Table 2A.** Comparison (M vs A) of manual (M) and automated (A) analysis of the lesion size in different cytoarchitectonic regions of the cerebral cortex in 9 mouse (#) with lateral –fluid –percussion induced traumatic brain injury. Data are expressed as a percentage of the area damaged for each cytoarchitectonic cortical subregion. Abbreviations: according to the mouse brain atlas of Franklin and Paxinos (2007). Orange shading indicates the absolute differences that were greater than 0. | | | | | | | | | | | | | | | | | | | | | | | | | | | |
| --- | --- | --- | --- | --- | --- | --- | --- | --- | --- | --- | --- | --- | --- | --- | --- | --- | --- | --- | --- | --- | --- | --- | --- | --- | --- | --- | --- |
|  | **#269** | | | **#271** | | | **#282** | | | **#288** | | | **#289** | | | **#291** | | | **#294** | | | **#297** | | | **#301** | | |
|  | **M** | **A** | **M vs A** | **M** | **A** | **M vs A** | **M** | **A** | **M vs A** | **M** | **A** | **M vs A** | **M** | **A** | **M vs A** | **M** | **A** | **M vs A** | **M** | **A** | **M vs A** | **M** | **A** | **M vs A** | **M** | **A** | **M vs A** |
| **Cg1** | 7 | 9 | 2 | 3 | 3 | 0 | 0 |  | 0 | 0 |  | 0 | 4 | 5 | 1 | 0 |  | 0 | 0 |  | 0 | 0 |  | 0 | 0 |  | 0 |
| **Cg2** | 0 |  | 0 | 0 |  | 0 | 0 |  | 0 | 0 |  | 0 | 0 |  | 0 | 0 |  | 0 | 0 |  | 0 | 0 |  | 0 | 0 |  | 0 |
| **RSD** | 51 | 54 | 3 | 16 | 19 | 3 | 1 | 1 | 0 | 18 | 20 | 2 | 42 | 45 | 3 | 19 | 22 | 3 | 35 | 38 | 3 | 30 | 34 | 4 | 0 |  | 0 |
| **RSGa** | 0 |  | 0 | 0 |  | 0 | 0 |  | 0 | 0 |  | 0 | 0 |  | 0 | 0 |  | 0 | 0 |  | 0 | 0 |  | 0 | 0 |  | 0 |
| **RSGb** | 0 | 1 | 1 | 0 |  | 0 | 0 |  | 0 | 0 |  | 0 | 0 |  | 0 | 0 |  | 0 | 0 |  | 0 | 0 |  | 0 | 0 |  | 0 |
| **RSGc** | 38 | 41 | 3 | 0 |  | 0 | 0 |  | 0 | 0 |  | 0 | 1 | 2 | 1 | 0 |  | 0 | 0 | 1 | 1 | 0 |  | 0 | 0 |  | 0 |
| **M1** | 28 | 29 | 1 | 41 | 43 | 2 | 7 | 9 | 2 | 27 | 29 | 2 | 31 | 32 | 1 | 3 | 4 | 1 | 6 | 7 | 1 | 9 | 10 | 1 | 0 |  | 0 |
| **M2** | 20 | 21 | 1 | 33 | 31 | 2 | 1 | 1 | 0 | 12 | 13 | 1 | 26 | 27 | 1 | 3 | 3 | 0 | 5 | 5 | 0 | 7 | 8 | 1 | 0 |  | 0 |
| **LPtA** | 100 | 100 | 0 | 100 | 100 | 0 | 100 | 100 | 0 | 100 | 100 | 0 | 100 | 100 | 0 | 100 | 100 | 0 | 100 | 100 | 0 | 100 | 100 | 0 | 47 | 44 | 3 |
| **MPtA** | 100 | 100 | 0 | 100 | 100 | 0 | 77 | 72 | 5 | 100 | 100 | 0 | 100 | 100 | 0 | 100 | 100 | 0 | 100 | 100 | 0 | 100 | 100 | 0 | 0 |  | 0 |
| **PTPR** | 100 | 100 | 0 | 100 | 100 | 0 | 100 | 100 | 0 | 100 | 100 | 0 | 100 | 100 | 0 | 100 | 100 | 0 | 100 | 100 | 0 | 100 | 100 | 0 | 100 | 100 | 0 |
| **S1** | 57 | 47 | 10 | 100 | 100 | 0 | 93 | 76 | 17 | 100 | 99 | 1 | 64 | 65 | 1 | 87 | 88 | 1 | 67 | 69 | 2 | 15 | 15 | 0 | 25 | 30 | 5 |
| **S1BF** | 66 | 67 | 1 | 73 | 76 | 3 | 40 | 41 | 1 | 74 | 75 | 1 | 40 | 41 | 1 | 48 | 49 | 1 | 43 | 43 | 0 | 31 | 32 | 1 | 40 | 41 | 1 |
| **S1DZ** | 64 | 64 | 0 | 84 | 83 | 1 | 46 | 50 | 4 | 73 | 74 | 1 | 48 | 48 | 0 | 18 | 23 | 5 | 32 | 32 | 0 | 30 | 28 | 2 | 18 | 23 | 5 |
| **S1DZ2** | 0 |  | 0 | 0 |  | 0 | 0 |  | 0 | 0 |  | 0 | 0 |  | 0 | 0 |  | 0 | 0 |  | 0 | 0 |  | 0 | 0 |  | 0 |
| **S1FL** | 77 | 78 | 1 | 88 | 88 | 0 | 49 | 51 | 2 | 80 | 81 | 1 | 77 | 78 | 1 | 2 | 3 | 1 | 13 | 14 | 1 | 19 | 20 | 1 | 2 | 4 | 2 |
| **S1HL** | 100 | 100 | 0 | 100 | 100 | 0 | 72 | 74 | 2 | 100 | 100 | 0 | 100 | 100 | 0 | 28 | 31 | 3 | 49 | 51 | 2 | 58 | 59 | 1 | 15 | 17 | 2 |
| **S1Sh** | 100 | 100 | 0 | 100 | 100 | 0 | 100 | 100 | 0 | 100 | 100 | 0 | 100 | 100 | 0 | 100 | 100 | 0 | 100 | 100 | 0 | 100 | 100 | 0 | 94 | 92 | 2 |
| **S1Tr** | 100 | 100 | 0 | 100 | 100 | 0 | 100 | 100 | 0 | 100 | 100 | 0 | 100 | 100 | 0 | 100 | 100 | 0 | 100 | 100 | 0 | 100 | 100 | 0 | 49 | 46 | 3 |
| **S1ULp** | 3 | 3 | 0 | 3 | 3 | 0 | 0 |  | 0 | 9 | 8 | 1 | 0 |  | 0 | 1 | 1 | 0 | 0 |  | 0 | 0 |  | 0 | 0 |  | 0 |
| **S2** | 0 |  | 0 | 1 | 1 | 0 | 0 |  | 0 | 3 | 3 | 0 | 0 |  | 0 | 0 | 0 | 0 | 0 |  | 0 | 0 |  | 0 | 0 |  | 0 |
| **SJ1S1** | 0 |  | 0 | 1 | 1 | 0 | 0 |  | 0 | 0 |  | 0 | 0 |  | 0 | 0 |  | 0 | 0 |  | 0 | 0 |  | 0 | 0 |  | 0 |
| **Au1** | 0 |  | 0 | 3 | 3 | 0 | 0 |  | 0 | 3 | 4 | 1 | 0 |  | 0 | 0 |  | 0 | 0 |  | 0 | 0 |  | 0 | 0 |  | 0 |
| **AuD** | 1 | 2 | 1 | 33 | 28 | 5 | 5 | 3 | 2 | 26 | 25 | 1 | 14 | 13 | 1 | 4 | 4 | 0 | 19 | 22 | 3 | 0 |  | 0 | 1 | 0 | 1 |
| **AuV** | 0 |  | 0 | 1 | 1 | 0 | 0 |  | 0 | 3 | 4 | 1 | 0 |  | 0 | 0 |  | 0 | 0 |  | 0 | 0 |  | 0 | 0 |  | 0 |
| **V1a** | 0 |  | 0 | 0 |  | 0 | 0 |  | 0 | 0 |  | 0 | 0 |  | 0 | 0 |  | 0 | 0 |  | 0 | 0 |  | 0 | 0 |  | 0 |
| **V1p** | 77 | 79 | 2 | 57 | 55 | 2 | 100 | 100 | 0 | 40 | 34 | 6 | 47 | 47 | 0 | 67 | 67 | 0 | 93 | 91 | 2 | 51 | 51 | 0 | 100 | 100 | 0 |
| **V1B** | 1 | 0 | 1 | 0 |  | 0 | 31 | 29 | 2 | 0 |  | 0 | 0 |  | 0 | 0 |  | 0 | 21 | 22 | 1 | 0 |  | 0 | 63 | 63 | 0 |
| **V1M** | 3 | 3 | 0 | 0 |  | 0 | 17 | 16 | 1 | 0 |  | 0 | 0 |  | 0 | 5 | 4 | 1 | 51 | 46 | 5 | 0 |  | 0 | 45 | 41 | 4 |
| **V2L** | 17 | 15 | 2 | 18 | 16 | 2 | 27 | 24 | 3 | 16 | 13 | 3 | 14 | 11 | 3 | 15 | 12 | 3 | 22 | 22 | 0 | 9 | 8 | 1 | 43 | 38 | 5 |
| **V2ML** | 42 | 46 | 4 | 35 | 37 | 2 | 51 | 56 | 5 | 22 | 23 | 1 | 30 | 32 | 2 | 50 | 53 | 3 | 66 | 71 | 5 | 34 | 37 | 3 | 54 | 60 | 6 |
| **V2MM** | 29 | 30 | 1 | 24 | 24 | 0 | 13 | 14 | 1 | 13 | 13 | 0 | 23 | 23 | 0 | 39 | 39 | 0 | 48 | 48 | 0 | 27 | 27 | 0 | 16 | 18 | 2 |
| **VIEnt** | 0 |  | 0 | 0 |  | 0 | 0 |  | 0 | 0 |  | 0 | 0 |  | 0 | 0 |  | 0 | 0 |  | 0 | 0 |  | 0 | 0 |  | 0 |
| **CEnt** | 0 |  | 0 | 0 |  | 0 | 0 |  | 0 | 0 |  | 0 | 0 |  | 0 | 0 |  | 0 | 0 |  | 0 | 0 |  | 0 | 0 |  | 0 |

| **Supplementary Table 2B.** Comparison (M vs A) of manual (M) and automated (A) analysis of the lesion size in different cytoarchitectonic regions of the cerebral cortex in 9 mouse (#) with lateral –fluid –percussion induced traumatic brain injury. Data are expressed as a percentage of the area damaged for each cytoarchitectonic cortical subregion. Abbreviations: according to the mouse brain atlas of Watson and Paxinos (2007). Green shading indicates the absolute differences that were greater than 0. | | | | | | | | | | | | | | | | | | | | | | | | | | | |
| --- | --- | --- | --- | --- | --- | --- | --- | --- | --- | --- | --- | --- | --- | --- | --- | --- | --- | --- | --- | --- | --- | --- | --- | --- | --- | --- | --- |
|  | **#329** | | | **#330** | | | **#331** | | | **#430** | | | **#434** | | | **#435** | | | **#436** | | | **#443** | | | **#444** | | |
|  | **M** | **A** | **M vs A** | **M** | **A** | **M vs A** | **M** | **A** | **M vs A** | **M** | **A** | **M vs A** | **M** | **A** | **M vs A** | **M** | **A** | **M vs A** | **M** | **A** | **M vs A** | **M** | **A** | **M vs A** | **M** | **A** | **M vs A** |
| **Cg1** | 0 |  | 0 | 0 |  | 0 | 0 |  | 0 | 0 |  | 0 | 0 |  | 0 | 0 |  | 0 | 0 |  | 0 | 0 |  | 0 | 0 |  | 0 |
| **Cg2** | 0 |  | 0 | 0 |  | 0 | 0 |  | 0 | 0 |  | 0 | 0 |  | 0 | 0 |  | 0 | 0 |  | 0 | 0 |  | 0 | 0 |  | 0 |
| **RSD** | 29 | 34 | 5 | 43 | 47 | 4 | 39 | 45 | 6 | 38 | 43 | 5 | 28 | 32 | 4 | 6 | 6 | 0 | 35 | 38 | 3 | 41 | 46 | 5 | 32 | 34 | 2 |
| **RSGa** | 0 |  | 0 | 0 |  | 0 | 0 |  | 0 | 0 |  | 0 | 0 |  | 0 | 0 |  | 0 | 0 |  | 0 | 0 |  | 0 | 0 |  | 0 |
| **RSGb** | 0 |  | 0 | 0 |  | 0 | 0 |  | 0 | 0 |  | 0 | 0 |  | 0 | 0 |  | 0 | 0 |  | 0 | 0 |  | 0 | 0 |  | 0 |
| **RSGc** | 0 | 0 | 0 | 2 | 3 | 1 | 2 | 3 | 1 | 1 | 2 | 1 | 0 |  | 0 | 0 |  | 0 | 3 | 5 | 2 | 2 | 3 | 1 | 0 |  | 0 |
| **M1** | 7 | 8 | 1 | 4 | 4 | 0 | 8 | 8 | 0 | 11 | 12 | 1 | 40 | 41 | 1 | 15 | 15 | 0 | 2 | 3 | 1 | 7 | 8 | 1 | 18 | 18 | 0 |
| **M2** | 6 | 7 | 1 | 3 | 4 | 1 | 5 | 6 | 1 | 9 | 10 | 1 | 23 | 24 | 1 | 4 | 5 | 1 | 1 | 2 | 1 | 5 | 6 | 1 | 8 | 10 | 2 |
| **LPtA** | 100 | 100 | 0 | 100 | 100 | 0 | 100 | 100 | 0 | 43 | 32 | 11 | 100 | 100 | 0 | 100 | 100 | 0 | 100 | 100 | 0 | 100 | 100 | 0 | 100 | 100 | 0 |
| **MPtA** | 100 | 100 | 0 | 100 | 100 | 0 | 100 | 100 | 0 | 100 | 100 | 0 | 100 | 100 | 0 | 76 | 70 | 6 | 100 | 100 | 0 | 100 | 100 | 0 | 100 | 100 | 0 |
| **PTPR** | 100 | 100 | 0 | 100 | 100 | 0 | 100 | 100 | 0 | 0 |  | 0 | 100 | 100 | 0 | 100 | 100 | 0 | 100 | 100 | 0 | 100 | 100 | 0 | 100 | 100 | 0 |
| **S1** | 68 | 47 | 21 | 0 |  | 0 | 65 | 65 | 0 | 0 |  | 0 | 81 | 67 | 14 | 0 |  | 0 | 26 | 15 | 11 | 1 |  | 1 | 62 | 54 | 8 |
| **S1BF** | 39 | 40 | 1 | 27 | 27 | 0 | 28 | 29 | 1 | 0 |  | 0 | 82 | 83 | 1 | 50 | 49 | 1 | 24 | 26 | 2 | 21 | 21 | 0 | 33 | 34 | 1 |
| **S1DZ** | 18 | 22 | 4 | 18 | 23 | 5 | 22 | 27 | 5 | 0 |  | 0 | 83 | 84 | 1 | 42 | 43 | 1 | 17 | 21 | 4 | 22 | 27 | 5 | 35 | 35 | 0 |
| **S1DZ2** | 0 |  | 0 | 0 |  | 0 | 0 |  | 0 | 0 |  | 0 | 0 |  | 0 | 0 |  | 0 | 0 |  | 0 | 0 |  | 0 | 0 |  | 0 |
| **S1FL** | 5 | 5 | 0 | 2 | 4 | 2 | 12 | 11 | 1 | 0 | 0 | 0 | 88 | 88 | 0 | 32 | 33 | 1 | 2 | 4 | 2 | 13 | 13 | 0 | 34 | 38 | 4 |
| **S1HL** | 50 | 48 | 2 | 33 | 35 | 2 | 51 | 52 | 1 | 59 | 59 | 0 | 100 | 100 | 0 | 75 | 77 | 2 | 29 | 31 | 2 | 50 | 52 | 2 | 100 | 92 | 8 |
| **S1Sh** | 100 | 100 | 0 | 100 | 100 | 0 | 100 | 100 | 0 | 48 | 43 | 5 | 100 | 100 | 0 | 100 | 100 | 0 | 100 | 100 | 0 | 100 | 100 | 0 | 100 | 100 | 0 |
| **S1Tr** | 100 | 100 | 0 | 100 | 100 | 0 | 100 | 100 | 0 | 35 | 41 | 6 | 100 | 100 | 0 | 100 | 100 | 0 | 100 | 100 | 0 | 100 | 100 | 0 | 100 | 100 | 0 |
| **S1ULp** | 0 |  | 0 | 0 |  | 0 | 0 |  | 0 | 0 |  | 0 | 11 | 13 | 2 | 0 |  | 0 | 0 |  | 0 | 0 |  | 0 | 0 |  | 0 |
| **S2** | 0 |  | 0 | 0 |  | 0 | 0 |  | 0 | 0 |  | 0 | 2 | 2 | 0 | 0 |  | 0 | 0 |  | 0 | 0 |  | 0 | 0 |  | 0 |
| **SJ1S1** | 0 |  | 0 | 0 |  | 0 | 0 |  | 0 | 0 |  | 0 | 2 | 3 | 1 | 0 |  | 0 | 0 |  | 0 | 0 |  | 0 | 0 |  | 0 |
| **Au1** | 0 |  | 0 | 0 |  | 0 | 1 | 0 | 1 | 0 |  | 1 | 1 | 0 | 1 | 0 |  | 0 | 0 |  | 0 | 0 |  | 0 | 0 |  | 0 |
| **AuD** | 0 |  | 0 | 0 |  | 0 | 17 | 19 | 2 | 0 |  | 17 | 3 | 2 | 1 | 0 |  | 0 | 0 |  | 0 | 0 |  | 0 | 2 | 3 | 1 |
| **AuV** | 0 |  | 0 | 0 |  | 0 | 0 |  | 0 | 0 |  | 0 | 5 | 3 | 2 | 0 |  | 0 | 0 |  | 0 | 0 |  | 0 | 0 |  | 0 |
| **V1a** | 0 |  | 0 | 5 | 8 | 3 | 0 |  | 0 | 0 |  | 0 | 0 |  | 0 | 0 |  | 0 | 0 |  | 0 | 0 |  | 0 | 0 |  | 0 |
| **V1p** | 100 | 100 | 0 | 100 | 100 | 0 | 100 | 100 | 0 | 0 |  | 0 | 83 | 84 | 1 | 67 | 68 | 1 | 51 | 51 | 0 | 84 | 83 | 1 | 69 | 69 | 0 |
| **V1B** | 12 | 13 | 1 | 93 | 87 | 6 | 68 | 68 | 0 | 0 |  | 0 | 23 | 23 | 0 | 0 |  | 0 | 0 |  | 0 | 0 |  | 0 | 0 |  | 0 |
| **V1M** | 43 | 38 | 5 | 100 | 100 | 0 | 81 | 80 | 1 | 4 | 6 | 2 | 58 | 58 | 0 | 0 |  | 0 | 13 | 12 | 1 | 8 | 8 | 0 | 6 | 7 | 1 |
| **V2L** | 21 | 18 | 3 | 31 | 31 | 0 | 37 | 25 | 12 | 0 |  | 0 | 10 | 9 | 1 | 4 | 4 | 0 | 8 | 6 | 2 | 13 | 11 | 2 | 16 | 13 | 3 |
| **V2ML** | 69 | 75 | 6 | 100 | 100 | 0 | 92 | 94 | 2 | 17 | 19 | 2 | 73 | 79 | 6 | 11 | 13 | 2 | 65 | 70 | 5 | 57 | 62 | 5 | 60 | 65 | 5 |
| **V2MM** | 44 | 44 | 0 | 61 | 54 | 7 | 50 | 51 | 1 | 34 | 36 | 2 | 48 | 48 | 0 | 3 | 4 | 1 | 49 | 49 | 0 | 47 | 47 | 0 | 32 | 33 | 1 |
| **VIEnt** | 0 |  | 0 | 0 |  | 0 | 0 |  | 0 | 0 |  | 0 | 0 |  | 0 | 0 |  | 0 | 0 |  | 0 | 0 |  | 0 | 0 |  | 0 |
| **CEnt** | 0 |  | 0 | 0 |  | 0 | 0 |  | 0 | 0 |  | 0 | 0 | 1 | 1 | 0 |  | 0 | 0 |  | 0 | 0 |  | 0 | 0 |  | 0 |

| **Supplementary Table 3. CLI arguments.** | |
| --- | --- |
| **Parameter** | **Description** |
| -m, --measurements | Path to the measurement file or to a folder containing measurements. |
| -o, --output-path | Output folder for mappings and area measurements. |
| -b, --border-color | Lesion border color as RGBA quadruplet, e.g. 0,0,0,255,1. Optional. |
| -f --fill-color | Lesion fill color as RGBA quadruplet, e.g. 0,0,0,255,1. Optional. |
| -w --border-width | Lesion border width as an integer value between 1 and 20. Optional. |
| -s, --border-style | Lesion border style. Accepted values are “solid”, “dashed” or “dotted”. Optional. |
| -d, --dpi | Output image dpi. Optional. |
| -t, --template | Path to the template used in mapping. Optional. |

| **Supplementary Table 4A.** Simulated M1, M2 and M3 measurements in different days used to generate lesion development video shown in **supplementary video 1** | | | | | | | | | | | | | | | | | | | |
| --- | --- | --- | --- | --- | --- | --- | --- | --- | --- | --- | --- | --- | --- | --- | --- | --- | --- | --- | --- |
| **Day 1** | | | | **Day 2** | | | | **Day 3** | | | | **Day 4** | | | | **Day 5** | | | |
| **MB** | **M1** | **M2** | **M3** | **MB** | **M1** | **M2** | **M3** | **MB** | **M1** | **M2** | **M3** | **MB** | **M1** | **M2** | **M3** | **MB** | **M1** | **M2** | **M3** |
| 0.740 | 3.613 | 0.118 | 4.248 | 0.740 | 3.554 | 0.236 | 4.189 | 0.740 | 3.495 | 0.355 | 4.130 | 0.740 | 3.436 | 0.473 | 4.071 | 0.740 | 3.377 | 0.591 | 4.012 |
| 0.500 | 3.491 | 0.289 | 4.067 | 0.500 | 3.346 | 0.577 | 3.922 | 0.500 | 3.202 | 0.866 | 3.778 | 0.500 | 3.058 | 1.154 | 3.634 | 0.500 | 2.914 | 1.443 | 3.490 |
| 0.260 | 2.782 | 0.201 | 5.093 | 0.260 | 2.682 | 0.402 | 4.993 | 0.260 | 2.581 | 0.604 | 4.892 | 0.260 | 2.481 | 0.805 | 4.792 | 0.260 | 2.380 | 1.006 | 4.691 |
| 0.020 | 3.160 | 0.312 | 4.075 | 0.020 | 3.004 | 0.625 | 3.919 | 0.020 | 2.847 | 0.937 | 3.762 | 0.020 | 2.691 | 1.250 | 3.606 | 0.020 | 2.535 | 1.562 | 3.450 |
| -0.460 | 2.699 | 0.405 | 4.689 | -0.460 | 2.496 | 0.810 | 4.486 | -0.460 | 2.294 | 1.215 | 4.284 | -0.460 | 2.091 | 1.620 | 4.081 | -0.460 | 1.889 | 2.026 | 3.879 |
| -0.700 | 2.643 | 0.414 | 4.441 | -0.700 | 2.436 | 0.827 | 4.234 | -0.700 | 2.229 | 1.241 | 4.027 | -0.700 | 2.022 | 1.655 | 3.820 | -0.700 | 1.815 | 2.069 | 3.613 |
| -1.060 | 2.512 | 0.418 | 4.166 | -1.060 | 2.303 | 0.835 | 3.957 | -1.060 | 2.094 | 1.253 | 3.748 | -1.060 | 1.885 | 1.671 | 3.539 | -1.060 | 1.676 | 2.089 | 3.330 |
| -1.340 | 2.932 | 0.530 | 4.075 | -1.340 | 2.667 | 1.061 | 3.810 | -1.340 | 2.402 | 1.591 | 3.545 | -1.340 | 2.137 | 2.121 | 3.280 | -1.340 | 1.872 | 2.652 | 3.015 |
| -1.700 | 2.443 | 0.482 | 4.306 | -1.700 | 2.202 | 0.964 | 4.065 | -1.700 | 1.961 | 1.446 | 3.824 | -1.700 | 1.720 | 1.928 | 3.583 | -1.700 | 1.479 | 2.411 | 3.342 |
| -1.940 | 2.417 | 0.473 | 5.035 | -1.940 | 2.181 | 0.947 | 4.799 | -1.940 | 1.944 | 1.420 | 4.562 | -1.940 | 1.707 | 1.894 | 4.325 | -1.940 | 1.471 | 2.367 | 4.089 |
| -2.180 | 2.400 | 0.482 | 4.948 | -2.180 | 2.159 | 0.963 | 4.707 | -2.180 | 1.918 | 1.445 | 4.466 | -2.180 | 1.677 | 1.927 | 4.225 | -2.180 | 1.436 | 2.409 | 3.984 |
| -2.460 | 2.850 | 0.506 | 4.701 | -2.460 | 2.597 | 1.012 | 4.448 | -2.460 | 2.344 | 1.517 | 4.195 | -2.460 | 2.091 | 2.023 | 3.942 | -2.460 | 1.839 | 2.529 | 3.690 |
| -2.540 | 2.922 | 0.446 | 5.035 | -2.540 | 2.699 | 0.892 | 4.812 | -2.540 | 2.476 | 1.338 | 4.589 | -2.540 | 2.253 | 1.784 | 4.366 | -2.540 | 2.030 | 2.230 | 4.143 |
| -2.700 | 3.487 | 0.312 | 4.812 | -2.700 | 3.331 | 0.625 | 4.656 | -2.700 | 3.175 | 0.937 | 4.500 | -2.700 | 3.019 | 1.249 | 4.344 | -2.700 | 2.863 | 1.562 | 4.188 |
| -2.800 | 3.318 | 0.192 | 4.848 | -2.800 | 3.222 | 0.383 | 4.752 | -2.800 | 3.127 | 0.575 | 4.657 | -2.800 | 3.031 | 0.766 | 4.561 | -2.800 | 2.935 | 0.958 | 4.465 |
| -3.080 | 4.202 | 0.082 | 3.822 | -3.080 | 4.161 | 0.163 | 3.781 | -3.080 | 4.120 | 0.245 | 3.740 | -3.080 | 4.080 | 0.326 | 3.700 | -3.080 | 4.039 | 0.408 | 3.659 |

| **Supplementary Table 4B. Simulated** M1, M2 and M3 measurements in different days used to generate lesion development video shown in **supplementary video 1** | | | | | | | | | | | | | | | | | | | |
| --- | --- | --- | --- | --- | --- | --- | --- | --- | --- | --- | --- | --- | --- | --- | --- | --- | --- | --- | --- |
| **Day 6** | | | | **Day 7** | | | | **Day 8** | | | | **Day 9** | | | | **Day 10** | | | |
| **MB** | **M1** | **M2** | **M3** | **MB** | **M1** | **M2** | **M3** | **MB** | **M1** | **M2** | **M3** | **MB** | **M1** | **M2** | **M3** | **MB** | **M1** | **M2** | **M3** |
| 0.74 | 3.317 | 0.709 | 3.952 | 0.74 | 3.258 | 0.827 | 3.893 | 0.74 | 3.199 | 0.946 | 3.834 | 0.74 | 3.14 | 1.064 | 3.775 | 0.74 | 3.081 | 1.182 | 3.716 |
| 0.5 | 2.769 | 1.732 | 3.345 | 0.5 | 2.625 | 2.02 | 3.201 | 0.5 | 2.481 | 2.309 | 3.057 | 0.5 | 2.336 | 2.597 | 2.912 | 0.5 | 2.192 | 2.886 | 2.768 |
| 0.26 | 2.279 | 1.207 | 4.59 | 0.26 | 2.179 | 1.408 | 4.49 | 0.26 | 2.078 | 1.61 | 4.389 | 0.26 | 1.978 | 1.811 | 4.289 | 0.26 | 1.877 | 2.012 | 4.188 |
| 0.02 | 2.379 | 1.874 | 3.294 | 0.02 | 2.223 | 2.187 | 3.138 | 0.02 | 2.066 | 2.499 | 2.981 | 0.02 | 1.91 | 2.812 | 2.825 | 0.02 | 1.754 | 3.124 | 2.669 |
| -0.46 | 1.686 | 2.431 | 3.676 | -0.46 | 1.484 | 2.836 | 3.474 | -0.46 | 1.281 | 3.241 | 3.271 | -0.46 | 1.079 | 3.646 | 3.069 | -0.46 | 0.876 | 4.051 | 2.866 |
| -0.7 | 1.608 | 2.482 | 3.406 | -0.7 | 1.402 | 2.896 | 3.2 | -0.7 | 1.195 | 3.31 | 2.993 | -0.7 | 0.988 | 3.723 | 2.786 | -0.7 | 0.781 | 4.137 | 2.579 |
| -1.06 | 1.467 | 2.506 | 3.121 | -1.06 | 1.259 | 2.924 | 2.913 | -1.06 | 1.05 | 3.342 | 2.704 | -1.06 | 0.841 | 3.759 | 2.495 | -1.06 | 0.632 | 4.177 | 2.286 |
| -1.34 | 1.607 | 3.182 | 2.75 | -1.34 | 1.341 | 3.712 | 2.484 | -1.34 | 1.076 | 4.242 | 2.219 | -1.34 | 0.811 | 4.773 | 1.954 | -1.34 | 0.546 | 5.303 | 1.689 |
| -1.7 | 1.238 | 2.893 | 3.101 | -1.7 | 0.997 | 3.375 | 2.86 | -1.7 | 0.756 | 3.857 | 2.619 | -1.7 | 0.515 | 4.339 | 2.378 | -1.7 | 0.274 | 4.821 | 2.137 |
| -1.94 | 1.234 | 2.84 | 3.852 | -1.94 | 0.997 | 3.314 | 3.615 | -1.94 | 0.76 | 3.787 | 3.378 | -1.94 | 0.524 | 4.261 | 3.142 | -1.94 | 0.287 | 4.734 | 2.905 |
| -2.18 | 1.195 | 2.89 | 3.743 | -2.18 | 0.955 | 3.372 | 3.503 | -2.18 | 0.714 | 3.854 | 3.262 | -2.18 | 0.473 | 4.335 | 3.021 | -2.18 | 0.232 | 4.817 | 2.78 |
| -2.46 | 1.586 | 3.035 | 3.437 | -2.46 | 1.333 | 3.541 | 3.184 | -2.46 | 1.08 | 4.046 | 2.931 | -2.46 | 0.827 | 4.552 | 2.678 | -2.46 | 0.574 | 5.058 | 2.425 |
| -2.54 | 1.807 | 2.676 | 3.92 | -2.54 | 1.584 | 3.122 | 3.697 | -2.54 | 1.361 | 3.568 | 3.474 | -2.54 | 1.138 | 4.014 | 3.251 | -2.54 | 0.915 | 4.46 | 3.028 |
| -2.7 | 2.707 | 1.874 | 4.032 | -2.7 | 2.55 | 2.186 | 3.875 | -2.7 | 2.394 | 2.498 | 3.719 | -2.7 | 2.238 | 2.811 | 3.563 | -2.7 | 2.082 | 3.123 | 3.407 |
| -2.8 | 2.839 | 1.15 | 4.369 | -2.8 | 2.743 | 1.341 | 4.273 | -2.8 | 2.648 | 1.533 | 4.178 | -2.8 | 2.552 | 1.724 | 4.082 | -2.8 | 2.456 | 1.916 | 3.986 |
| -3.08 | 3.998 | 0.489 | 3.618 | -3.08 | 3.957 | 0.571 | 3.577 | -3.08 | 3.917 | 0.652 | 3.537 | -3.08 | 3.876 | 0.734 | 3.496 | -3.08 | 3.835 | 0.815 | 3.455 |

**Supplementary Figure 1.** Unfolded maps generated using the automated web application (red dashed outline) superimposed on the manually generated map (light green shading) from the remaining 17 cases that were not shown in Fig.5.

**Supplementary Figure 2.** A flow diagram demonstrating the normalization, translation, interpolation and quantification processes implemented by the web application in lesion mapping.

**Supplementary video 1.** Animated video depicting lesion development over time. Video was produced using hypothetical time series data in supplementary Table 4.
