# Supplementary figures and images for: Web Application for Quantification of Traumatic Brain Injury-Induced Cortical Lesions in Adult Mice
Source: Neuroinformatics. 2019 Dec 4;18(2):307–17. doi: 10.1007/s12021-019-09444-9 (PMC7083813; doi:10.1007/s12021-019-09444-9)

## Slide 1
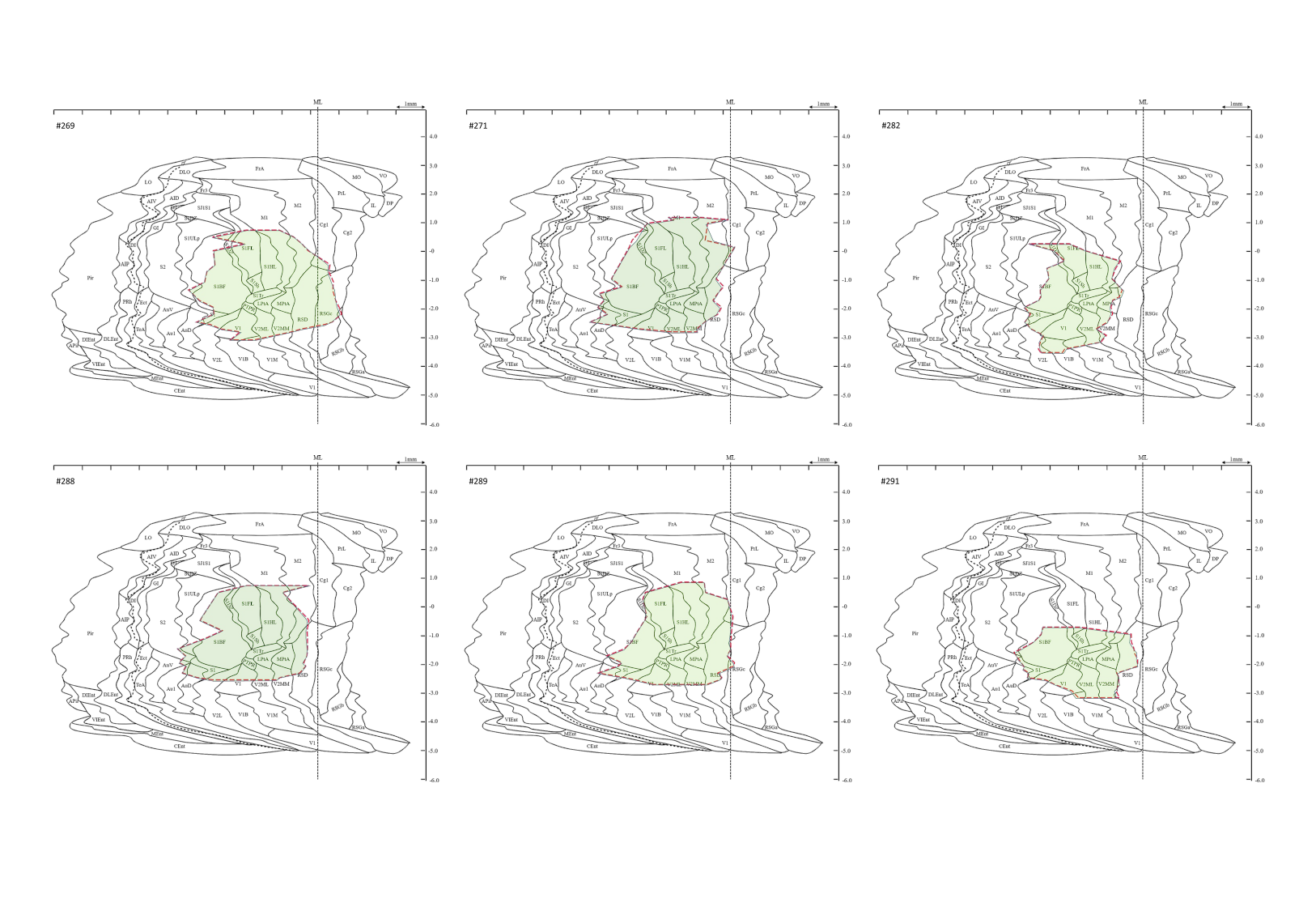

## Slide 2
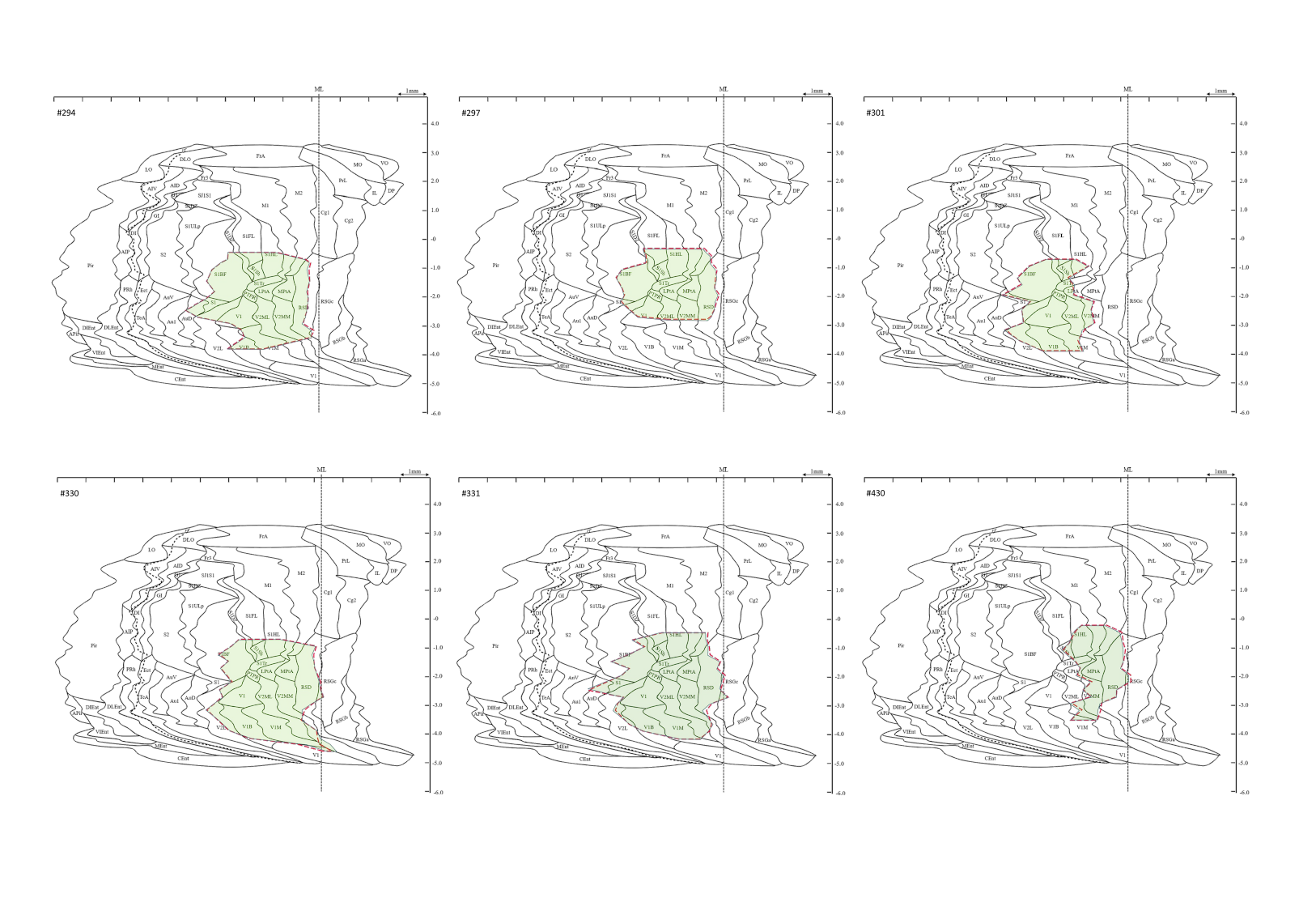

## Slide 3
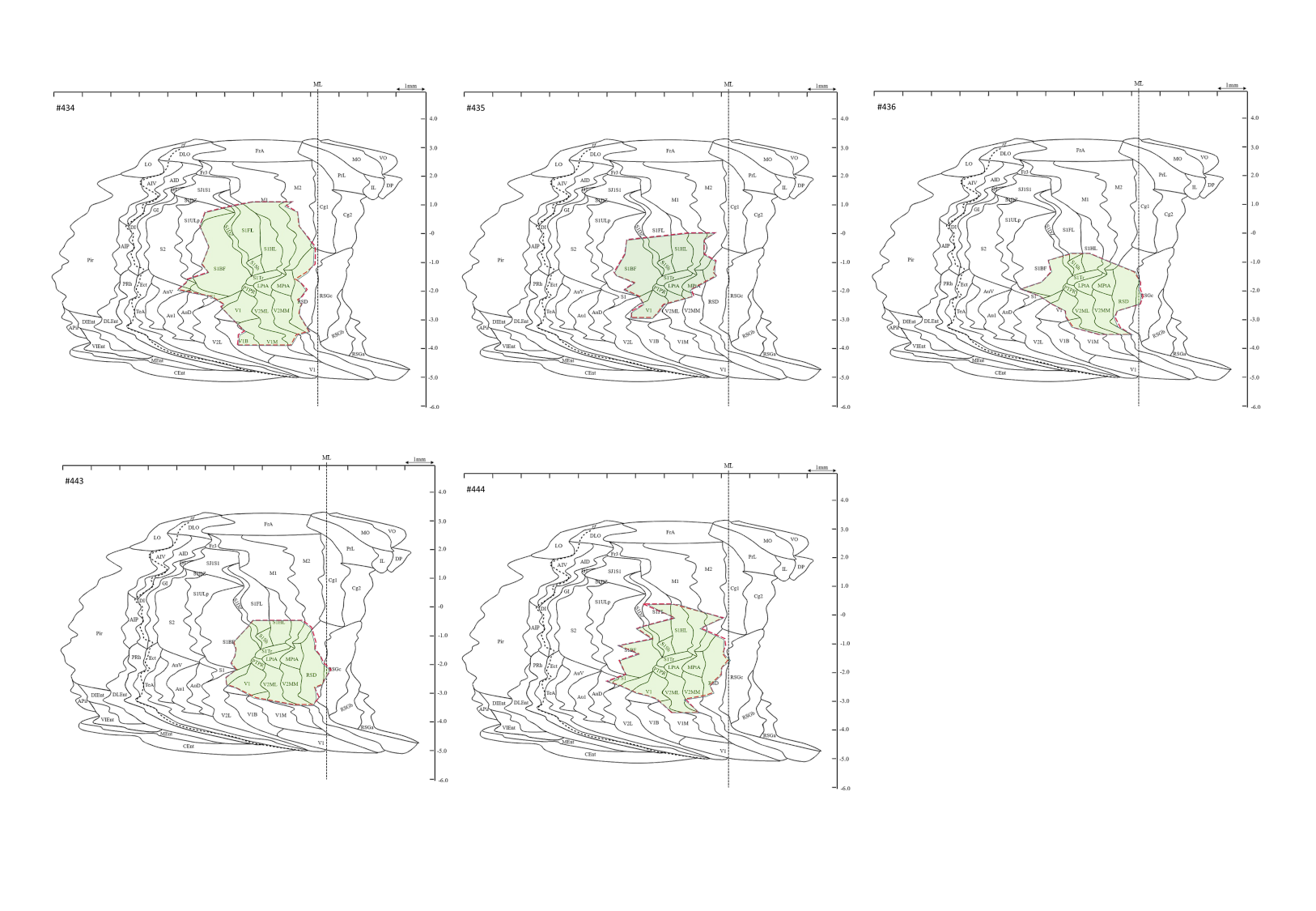

Supplement: Supplementary file 2 — (PPTX 11813 kb) [file 12021_2019_9444_MOESM2_ESM.pptx]

# Normalization

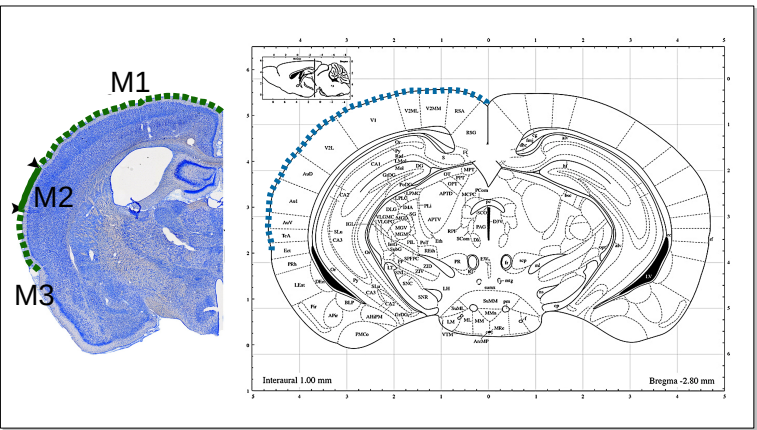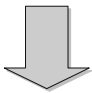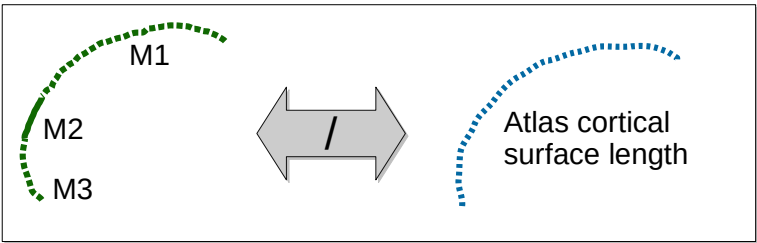

m/a Measurement vs. atlas ratio

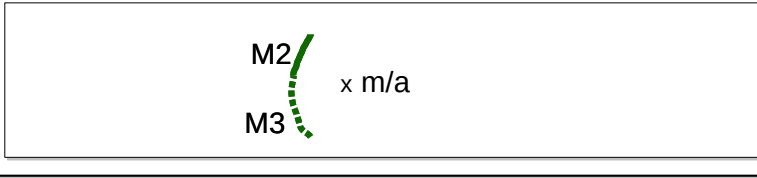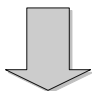

# Translation

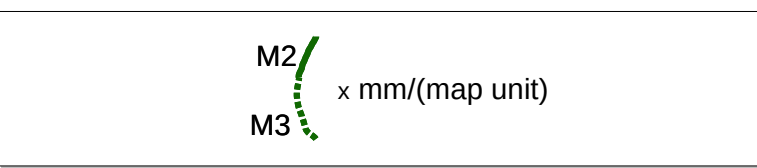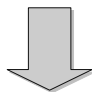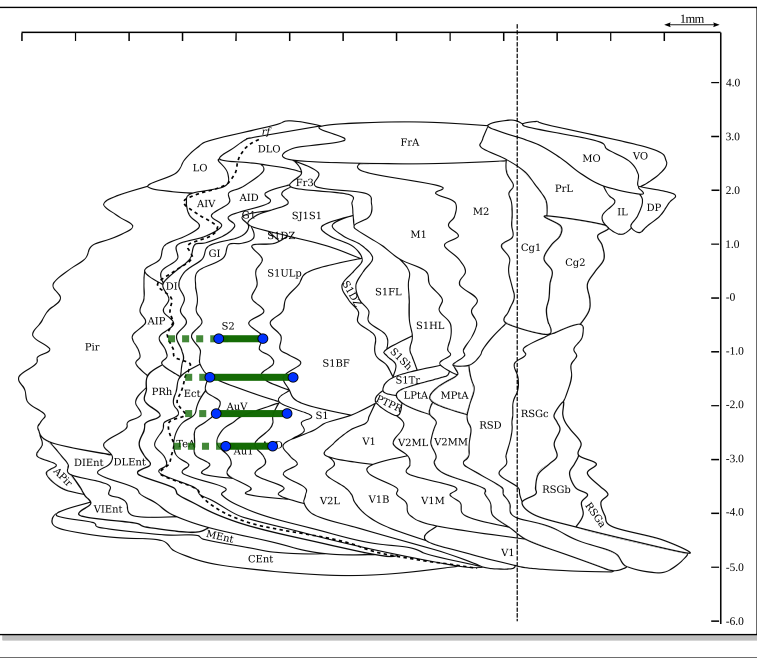

# Quantification

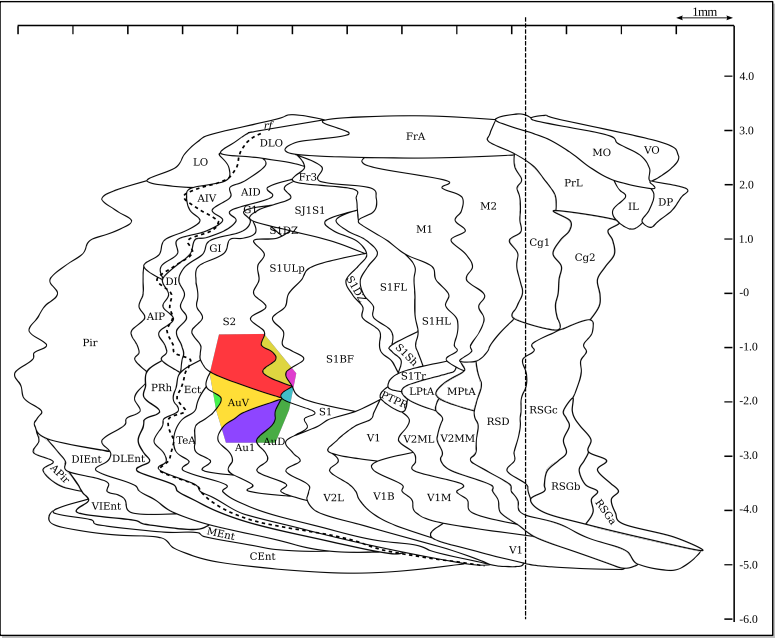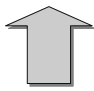

# Interpolation

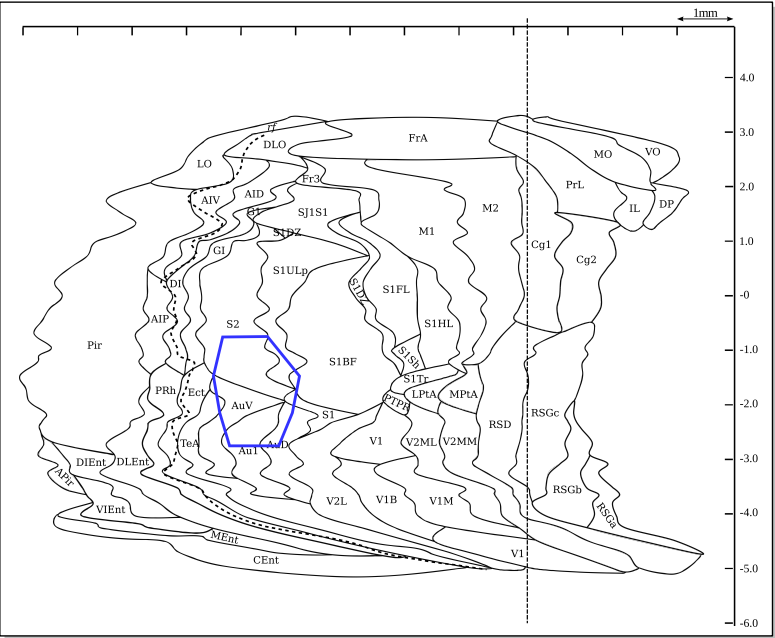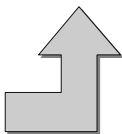

Supplement: Supplementary file 3 — (PDF 9600 kb) [file 12021_2019_9444_MOESM3_ESM.pdf]
